# Location-Dependent Effects of Inhibition on Local Spiking in Pyramidal Neuron Dendrites

**DOI:** 10.1371/journal.pcbi.1002550

**Published:** 2012-06-14

**Authors:** Monika Jadi, Alon Polsky, Jackie Schiller, Bartlett W. Mel

**Affiliations:** 1 Department of Biomedical Engineering, University of Southern California, Los Angeles, California, United States of America; 2 Technion Medical School, Haifa, Israel; 3 Neuroscience Graduate Program, University of Southern California, Los Angeles, California, United States of America; École Normale Supérieure, College de France, CNRS, France

## Abstract

Cortical computations are critically dependent on interactions between pyramidal neurons (PNs) and a menagerie of inhibitory interneuron types. A key feature distinguishing interneuron types is the spatial distribution of their synaptic contacts onto PNs, but the location-dependent effects of inhibition are mostly unknown, especially under conditions involving active dendritic responses. We studied the effect of somatic vs. dendritic inhibition on local spike generation in basal dendrites of layer 5 PNs both in neocortical slices and in simple and detailed compartmental models, with equivalent results: somatic inhibition divisively suppressed the amplitude of dendritic spikes recorded at the soma while minimally affecting dendritic spike thresholds. In contrast, distal dendritic inhibition raised dendritic spike thresholds while minimally affecting their amplitudes. On-the-path dendritic inhibition modulated both the gain and threshold of dendritic spikes depending on its distance from the spike initiation zone. Our findings suggest that cortical circuits could assign different mixtures of gain vs. threshold inhibition to different neural pathways, and thus tailor their local computations, by managing their relative activation of soma- vs. dendrite-targeting interneurons.

## Author Summary

Establishing how inhibitory neurons shape the computing functions of neural circuits is crucial to understanding both normal function and dysfunction in the human brain. It has been known for over a century that different classes of inhibitory interneurons project to different sub-regions of the neurons they contact – some primarily target cell bodies, others the dendrites, still others the axon. It remains poorly understood, however, how these different projection patterns influence synaptic integration in the target neuron populations. By providing new data from intracellular recordings in brain slices, and a simple but powerful model of the location-dependent effects of inhibition on dendritic spike generation, our study (1) demonstrates the importance of the absolute and relative locations of excitatory and inhibitory inputs to pyramidal neurons, the principal cells of the cerebral cortex, and (2) helps to establish a more solid theoretical understanding of these complex integrative phenomena. As high resolution mapping of the cortical “connectome” becomes available in the coming years, our work will be helpful in interpreting the computing functions of cortical tissue both at the single neuron and circuit levels.

## Introduction

The sensory, motor, and cognitive functions of neocortical circuits depend critically on synaptic interactions between pyramidal neurons (PN), the principal excitatory neurons of the neocortex, and a multitude of inhibitory interneuron types [1], [2]. Understanding the “arithmetic” governing these excitatory-inhibitory interactions at the single neuron level is therefore crucial to our understanding of neocortical function [3]–[14]. The problem is complex given the diversity of interneurons, which can be divided into subtypes based on morphology, firing pattern, expression of calcium-binding proteins and neuropeptides, and properties of input and output synapses [1], [2].

One of the most salient features distinguishing cortical interneurons, however, is the spatial distribution of the synaptic contacts they form onto their PN targets. For example, basket cells target the soma and peri-somatic region [15], [16], double bouquet cells target non-apical dendritic shafts and spines while avoiding the soma [15], [17], Martinotti cells target apical tuft dendrites [15], and chandelier cells target axon initial segments [18].

Several studies have explored the location-dependence of excitatory-inhibitory (E-I) interactions under passive conditions in a variety of cell types, mainly focusing on the effectiveness of inhibition at different locations relative to an excitatory input. In the first systematic study of this issue, Koch et al. [12] showed in a retinal ganglion cell model that inhibition was most effective at reducing somatic EPSPs when placed on the path to the soma, and was much less effective at distal locations or on other branches. Consistent with this, Hao et al. [4] showed that the divisive interaction between excitation and inhibition in CA1 pyramidal cells falls off steeply as the inhibition moves distally relative to the site of excitation, but remains relatively constant as the inhibition moves along the path to the soma. Liu (2004) also reported an asymmetric decay of inhibitory effectiveness moving away from a site of excitation in the dendrites of cultured hippocampal neurons, but with a slightly greater inhibitory effect just distal to the excitation. Vu and Krasne [10] distinguished the effects of proximal and distal inhibition in more general terms, calling distal inhibition “relative”, in the sense that no matter how large an inhibitory conductance is applied, it can be overcome by increasing the level of excitation. By contrast, proximal inhibition (including on-the-path and somatic inhibition) produces an “absolute” reduction in the magnitude of the somatic EPSP that cannot be overcome by any amount of distal excitation.

Much less is known about E-I synaptic location effects under “active” response conditions, that is, when PN dendrites are driven to generate local spikes [19]–[27]. A tentative conclusion based on previous modeling studies is that local spikes in the thin dendrites of pyramidal neurons are particularly susceptible to interruption or outright block by even small amounts of properly timed dendritic inhibition, whereas somatic inhibition is almost completely ineffective at blocking dendritic spikes [13], [28]; a recent experimental study in CA1 pyramidal cells has come to similar conclusions [29]. Beyond these few observations about inhibitory “effectiveness”, many uncertainties remain as to how inhibitory synapses at different locations on the cell differentially and quantitatively affect the dendritic spike generation process, or the conduction of dendritic spikes to the soma once they do occur. To help clarify these issues, we performed intracellular recordings in brain slices to quantify the effects of the location of inhibition on local spike generation in basal dendrites of layer 5 PNs. We then characterized the mechanisms underlying the E-I location effects using both detailed and simplified compartmental modeling approaches.

## Results

### Location dependent E-I effects: experimental data from basal dendrites of layer 5 pyramidal neurons

**Figure 1:**
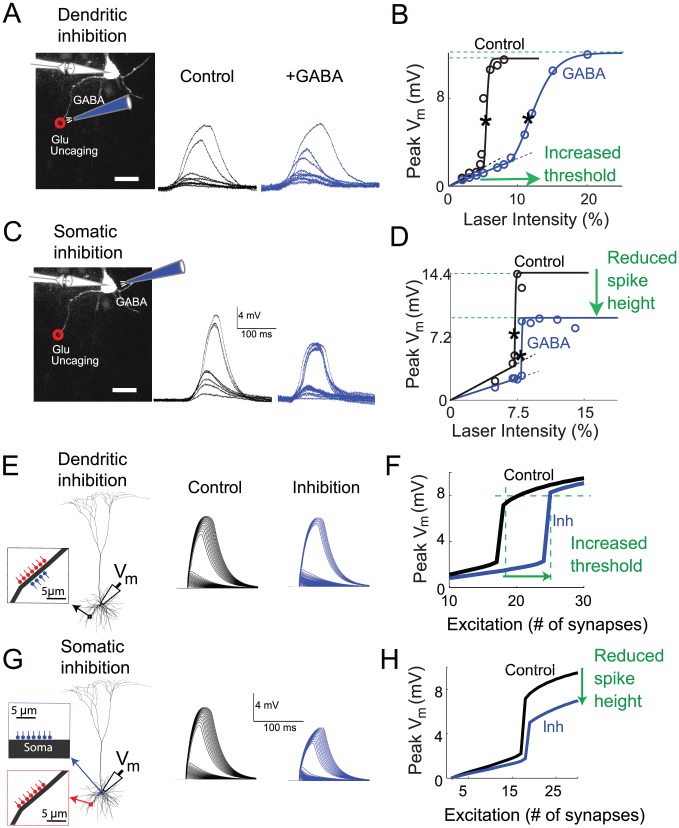
Inhibitory location effects: electrophysiological recordings from brain slices and detailed compartmental model. (A, C) Whole-cell somatic recording were carried out in a layer 5 pyramidal cell. Excitation was provided by UV laser uncaging of glutamate at a site 150 µm from the soma in a basal dendrite. Inhibition was delivered via GABA iontophoresis at the same site (A) or at the soma (C). Excitation was delivered at least 10 ms after the iontophoresis. Black traces show control case without inhibition, blue traces are in the presence of inhibition. (B, D) Input-output curves for peak somatic depolarization as a function of laser intensity. Spike thresholds indicated by asterisks were computed from sigmoidal fits to the i/o curves (see Methods); spike heights were computed from asymptotic values of sigmoidal fits, indicated by horizontal dashed lines. (E, G) Voltage traces at the soma generated by a detailed compartmental model of a layer-5 pyramidal cell. Excitatory synapses (NMDA+AMPA) were placed on a single basal dendrite 125 µm from the soma and inhibitory (GABA_A_) synapses were either co-localized with the excitation (E) or placed at the soma (G). Line colors and dashing are as in a–d. (F, H) Input-output curves for compartmental model as a function of activated excitatory synapses. Each excitatory synapse in this experiment had 6 nS peak AMPA conductance. Excitatory synapses with 1.5 nS peak AMPA conductance with similar distribution of density along the dendrite gave similar results. For the cases shown, peak inhibitory conductance was 10 nS in case of dendritic inhibition case and 90 nS in case of somatic inhibition.

We tested the effects of the location of inhibition in experiments in neocortical somatosensory slices. Whole cell somatic recordings were made from layer 5 pyramidal neurons. Excitation was delivered to a dendritic site ranging from 85 to 200 µm from the soma either by electrical stimulation or glutamate uncaging (Figure 1). Inhibition was applied via GABA iontophoresis either near the dendritic site of excitation (Figure 1A) or at the soma (Figure 1C). Due to the slow rate of onset of the inhibitory response (Figure S1A), the excitation (whether by glutamate uncaging or electrical stimulation) followed the GABA iontophoresis pulse by 10–200 ms (Figure S1C).

#### Effects of co-localized dendritic inhibition

**Figure 2:**
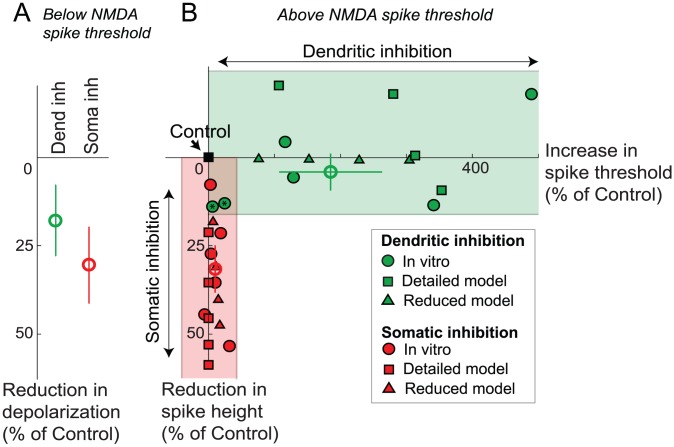
Summary of location effects of inhibition. (A) % reduction in somatic depolarization caused by dendritic vs. somatic inhibition at stimulus levels subthreshold for NMDA spike initiation, averaged over subthreshold part of i/o curves like those shown in Figure 1 B,D. Bars shown are standard errors. (B) Scatter plot showing changes in NMDA spike threshold (x-axis) and height (y-axis) in response to dendritic (green symbols) and somatic (red symbols) inhibition, expressed as joint % change in spike height and threshold relative to no-inhibition control (black square at origin). Peak conductance for dendritic inhibition cases shown here was 10, 20, 30, and 40 nS, while that for somatic inhibition was 30, 60, 90, 120 and 150 nS. Each excitatory synapse in this experiment had 6 nS peak AMPA conductance. Excitatory synapses with 1.5 nS peak AMPA conductance with similar distribution of density along the dendrite gave similar results. The figure includes data from *in vitro* experiments (circles), detailed compartmental model (squares) and the reduced (2-compartment) steady state model (triangles). Open circles show the means of the respective *in vitro* data. Green and red shaded regions highlight the predominance of threshold elevation in cases of dendritic inhibition, and height suppression in cases of somatic inhibition.

To examine the effects of inhibition co-localized with the excitatory stimulus, we applied increasing levels of dendritic excitation until a local spike was evoked, both under control conditions without inhibition (Figure 1A, black traces), and paired with co-localized inhibition (Figure 1A, blue traces). Input-output curves for the traces in Figure 1A are shown in Figure 1B, plotting peak somatic voltage responses vs. stimulation intensity. For low levels of excitation that remained subthreshold for local spike generation (first 3 data points in Figure 1B), dendritic inhibition reduced somatic EPSPs by 25.8%, leading to a corresponding reduction in the initial slope of the i/o curve relative to the control condition (compare black and blue dashed lines in Figure 1B). On average across cells, initial i/o curve slopes were reduced by 19±10% compared to their pre-inhibition values (p = 0.12, Student's t-test, n = 6; Figure 2a, open green circle).

When higher levels of excitation were applied, local dendritic spikes were generated (Figure 1A,B). Based on earlier studies, we refer to these responses as NMDA spikes reflecting the dominant contribution of NMDA currents to the regenerative process [24], [30]. We defined two features of the input-output curves. First, the spike threshold (marked by asterisks in Figure 1B) was defined as the level of excitation (plotted on the x-axis) at the steepest slope of a sigmoidal fit to the input-output data (Figure 1B, solid curves). Second, spike height (marked by horizontal dashed lines in Figure 1B) was defined as the voltage asymptote of the sigmoidal fit to the input-output data (Figure 1B). In the example shown in Figure 1A,B, NMDA spike height in the presence of inhibition showed a 4.4% increase compared to the control case without inhibition. On average, dendritic inhibition produced a non-significant change in spike height (−4±5.2%, p = 0.48, Student's t-test, n = 6). In contrast to the lack of effect on spike height, dendritic inhibition substantially increased the spike threshold (216% of control in the example of Figure 1B and 284±78% on average, p = 0.065, Student's t-test, n = 6). Figure 2 shows the joint changes in spike threshold and spike height in the 6 cases of co-localized dendritic inhibition (green circles).

#### Effects of somatic inhibition

Like dendritic inhibition, somatic inhibition also suppressed subthreshold EPSP peaks recorded at the soma (39% reduction in the case of Figure 1C,D and 30±10.8% on average; p = 0.04, Student's t-test, n = 6; Figure 2A, open red circle). When dendritic spikes were generated, however, the effects of somatic inhibition were nearly opposite to those seen for dendritic inhibition. As shown in the case of Figure 1A,C, somatic inhibition reduced the magnitude of the dendritic spike recorded at the soma by 35.4%, while causing only a slight elevation in the threshold for spike initiation (10% increase relative to control, Figure 1D). On average, NMDA spike amplitude recorded at the soma was reduced by 31±7% (p = 0.005, Student's t-test, n = 6). In contrast, the threshold level of dendritic excitation needed to generate spikes was not significantly changed by somatic inhibition (+10±5%, p = 0.1229, Student's t-test, n = 6). The joint effects of somatic inhibition on spike height and threshold are shown in Figure 2B (red circles).

In summary, the strong suppressive effect of somatic inhibition on NMDA spike height coupled with its minimal effect on spike threshold is reflected by the primarily vertical distribution of the red circles close to the y-axis in Figure 2B. In contrast, the strong effect of dendritic inhibition on spike threshold coupled with its non-effect on spike height is captured by the primarily horizontal distribution of green circles straddling the x-axis.

### A detailed compartmental model shows a similar pattern of inhibitory location effects

The slow and broad spatiotemporal profile of glutamate uncaging in our experimental protocol, although reasonably matched to NMDA channel kinetics, was much slower than the activation times of AMPA receptor-mediated synaptic currents. Likewise, the slow time course of GABA iontophoresis (Figure S1A) could have worked against the precise localization of activated GABA receptors in the membrane. To assess whether our main results would hold for more realistic synaptic time courses and precise input localization, we repeated the inhibitory location experiments in a biophysically detailed 268-compartment model of a reconstructed layer-5 pyramidal cell. The membrane potential dynamics of each compartment were calculated using the NEURON simulation environment (see Methods). Excitation was provided by tightly-spaced synapses with mixed NMDA-AMPA conductances placed on a basal dendrite 125 µm from the cell body. Inhibitory synapses were modeled as GABA_A_-type conductances (based on [31]) which caused comparable input resistance changes at the soma as were seen in the experiments (Figure S2). The inhibitory synapses were either co-localized with the excitatory synapses (Figure 1E) or placed at the soma (Figure 1G). In addition to synaptic and leak channels, the dendrites contained low concentrations of Hodgkin-Huxley-type Na^+^ and K^+^ channels adjusted to match dendritic recordings in [32].

The location-dependent effects of inhibition in the compartmental model were very similar to the experimental results described above. Dendritic inhibition substantially increased the threshold level of excitation needed to initiate an NMDA spike, but had little effect on spike height as measured at the soma (Figure 1E,F). In contrast, when inhibitory synapses were placed at the soma, the NMDA spike threshold was only slightly increased, whereas the spike height at the soma was substantially reduced (Figure 1G,H). The suppressive effect of somatic inhibition on spike height was clearly divisive (Figure S8). Several cases with varying levels of inhibition are shown in Figure 2B (green and red squares). We found the effects were robust over a physiologically realistic range of time courses and delays [33] in excitatory and inhibitory conductances (Figure S7). One difference in the modeling results compared to the experimental data was a paradoxical increase in NMDA spike height seen in the model in the presence of dendritic inhibition (Figure 1B,F, Figure 2B). Factors that could explain this and the lack of a similar increase in spike height in the slice data are considered in the Discussion. The main effects seen in the compartmental model did not change when the simulations were repeated with voltage-dependent Na^+^ and K^+^ channels blocked in the dendrites, leaving NMDA channels as the sole source of regenerative current in the cell membrane (results not shown). In contrast, blocking NMDA channels eliminated local spikes altogether in the model, as in previous experimental studies [24], [32], [34], suggesting that the location effects we observed experimentally can be accounted for by interactions between NMDA currents and the passive cable properties of PN dendrites.

### Invariance of dendritic spike height

**Figure 3:**
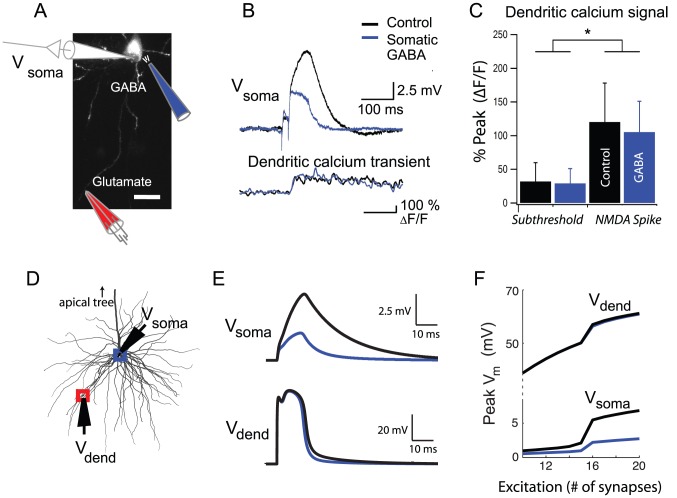
Dendritic spike height is not affected by somatic inhibition. (A) Experimental setup for testing somatic inhibition. Red electrode shows dendritic site of stimulation, blue electrode shows somatic site of GABA iontophoresis. (B) Voltage traces (top) and dendritic calcium signal (bottom) for control case (black) and with somatic inhibition (blue). (C) Bar plots compare dendritic calcium signal peaks (control: black, GABA: blue) for EPSPs that were both subthreshold and suprathreshold to NMDA spikes. (D) Morphology and stimulation set up in detailed compartmental model. Red square indicates location of excitatory synapses on a single dendrite, while the blue square indicates somatic location of inhibitory synapses. (E) Membrane potential at the soma and dendritic location for increasing levels of excitation (6 nS per synapse). Black traces indicate control, while blue traces indicate co-stimulation of somatic inhibitory synapses (peak conductance = 90 nS). (F) I/O curves at the soma and at the dendritic location for peak Vm for control (black) and somatic inhibition (blue).

A key difference between dendritic and somatic inhibition conditions was the observation of full-height spikes at the soma under increasing levels of dendritic inhibition, in contrast to a gradual reduction in the peak response at the soma under increasing levels of somatic inhibition (Figure 2). The graded suppression of peak responses at the soma by somatic inhibition could have been due to a gradual suppression of peak responses at the distal site of spike generation, reflecting a gradual weakening of NMDA current regenerativity. Alternatively, the dendritic spike could have remained constant in height locally in the dendrite, with the suppression explained by a greater attenuation of the voltage signal transferred from the dendrite to the soma. To distinguish these cases, we performed simultaneous voltage recordings at the soma and calcium imaging in the activated dendrite, measuring peak calcium transients with the indicator OGB-1 (Figure 3A). Calcium transients in the presence and absence of somatic inhibition were indistinguishable (control: 120±57%, GABA: 105±46%; non-significant with ANOVA), and significantly higher than those associated with just-subthreshold levels of excitation (p<0.01, Figure 3B,C), suggesting that the regenerative capacity at the dendritic site was unaltered by the presence of somatic inhibition (Figure 3B,C). Given uncertainties in the interpretation of calcium transients as surrogates for membrane potential, however, we directly measured dendritic voltages in compartmental simulations under comparable experimental conditions (Figure 3D,E). Consistent with the lack of change in the calcium transients seen in the experiments, dendritic spike heights in the model were also virtually unchanged by somatic inhibition, despite the substantial spike height reduction measured at the soma (Figure 3E,F). Thus, the experimental and modeling data were both consistent with invariant spike height for either location of inhibition.

### Location effects are captured by a time-invariant 2-compartment model

**Figure 4:**
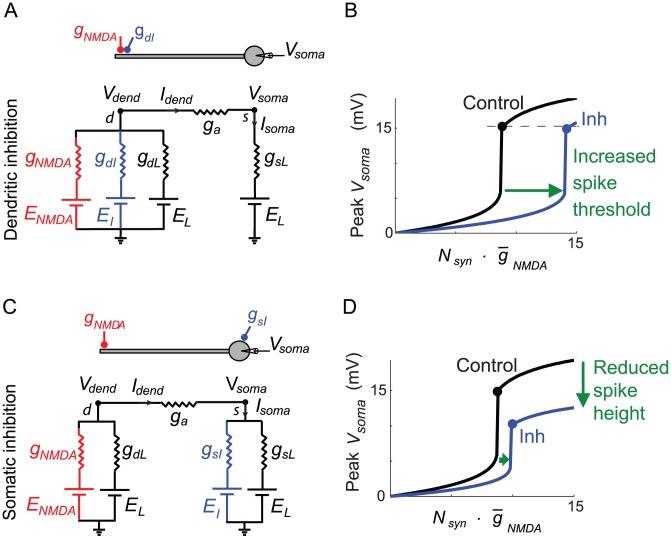
Dendritic vs. somatic inhibition in a 2-compartment model. (A,C) Two-compartment models (see Methods for details) contained an NMDA conductance in the dendrite (node *d*) scaled by *N_syn_* and an inhibitory conductance either in the dendrite (A) or soma (C). (B, D) Input-output curves in the somatic compartment (node *s*) with and without inhibition. Curves reproduce main features of input-output curves from experiments and detailed compartmental modeling results (Figure 1).

The similarity of our experimental and modeling data, despite the much slower time course of synaptic action in our slice experiments compared to the compartmental simulations, suggested that inhibitory location effects might depend mainly on the voltage-dependence of the NMDA conductance rather than its time course. To test this hypothesis and to probe the biophysical mechanisms underlying the location-dependent effects we had observed, we analyzed the input-output behavior of a time-invariant 2-compartment model as in Vu and Krasne [10], but where a voltage-dependent NMDA conductance replaced the AMPA-like conductance used in [9] as the source of dendritic excitation (Figure 4A,C). The equations used to model the NMDA conductance and to calculate NMDA spike threshold and height are described in the Methods.

We plotted the response of the model neuron to an increasing number of activated NMDA channels (*N_syn_*), covering the range from subthreshold to suprathreshold responses. We asked whether such a simplified model, which captures the spatial separation of the NMDA spike initiation zone in the dendrite and the soma, but suppresses many details including all temporal dynamics, could replicate the location-dependent effects of inhibition on NMDA spike generation described above. We found the 2-compartment model closely matched both the slice data and the results of our detailed compartmental simulations, including the reduction in slope in the subthreshold response range (Figure 4B,D), the invariant spike height in the dendritic compartment regardless of the location of inhibition, the relatively large elevation in spike threshold by dendritic inhibition (Figure 4B), and the slightly elevated threshold but strongly suppressed spike height (in the somatic compartment) in the case of somatic inhibition (Figure 4D). The effects of inhibition in the simplified model are summarized in Figure 2B (green and red triangles).

#### Principles underlying the inhibitory location effects

Given that the 2-compartment model contained only 5 time-invariant conductances (compared to hundreds of coupled nonlinear differential equations underlying the detailed compartmental model) an analysis of its steady state solutions allowed the effects of dendritic vs. somatic inhibition seen in the slice data to be explained based on 3 simple principles:

1. *Inhibition anywhere increases the leak conductance at the site of spike initiation, which proportionally raises the NMDA spike threshold. But once triggered, the NMDA spike is invariant in height at the site of initiation regardless of the effective total leak conductance that had to be overcome.*

**Figure 5:**
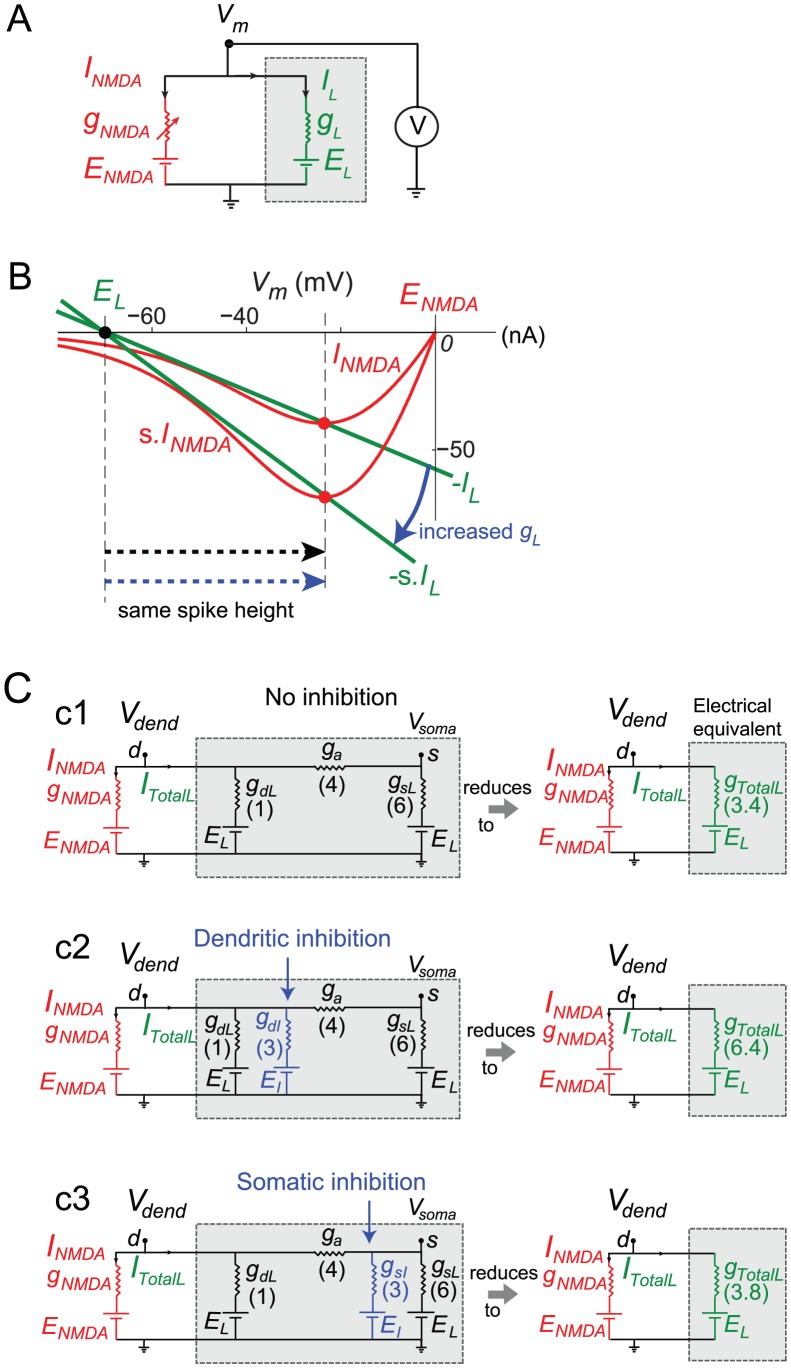
Mechanisms underlying stereotypical NMDA spike height. (A) A single voltage compartment containing NMDA and leak conductances. (B) I–V curves for two levels of leak and threshold NMDA conductances for the circuit shown in A. Increasing leak scales the leak I–V curve (shown in green, sign reversed and reflected below the x-axis). Levels of NMDA conductance shown (red I–V curves) correspond to two different values of channels *N_syn_* in equation 1 and were just suprathreshold for NMDA spike generation in control and increased leak cases. Spike heights are same in two cases (black and blue dashed arrows). (C) Equivalent circuits from perspective of dendritic compartment (node *s*) *for* cases with no inhibition (c1), with dendritic inhibition (c2) or with somatic inhibition (c3), (*E_L_* = *E_I_*). Shaded grey areas indicate all conductances contributing to total leak from the perspective of the dendritic compartment.

Details. In a single electrical compartment (Figure 5A), increasing the total leak conductance by activating shunting inhibition produces a multiplicative scaling of the leak I–V curve – compare 2 green curves in Figure 5B. To generate an NMDA spike in the presence of an increased total leak, an original just-suprathreshold NMDA conductance (see Figure S3E) must be scaled up by the same factor as the increase in total leak conductance to re-obtain the threshold condition (see 2 red curves representing NMDA I–V curves skimming just below two green curves in Figure 5B). Importantly, under this co-scaling of NMDA and total leak conductances, the voltage at the intersection of the NMDA and leak I–V curves (red dots) remains unchanged, which predicts a constant NMDA spike height regardless of the level of inhibition (see Text S1 for further explanation).

2. *Dendritic inhibition increases the dendritic leak conductance much more than does somatic inhibition, and thus leads to a much larger increase in the dendritic spike threshold.*

Details. From the perspective of the dendritic compartment where a local spike is initiated, inhibition at *any* location – whether locally in the dendrite or remotely at the soma – produces an increase in effective leak conductance in the dendritic compartment (see Figure 5C). However, the strength of the effect depends heavily on the location of inhibition. Electrical equivalent circuits from the dendritic perspective are shown in Figure 5C for cases without inhibition (C1), with dendritic inhibition (C2), and with somatic inhibition (C3). The equivalent circuits are structurally identical, differing only in the expressions for *g_TotalL_* representing the total effective leak conductance that must be overcome to reach the NMDA spike threshold in each case. As shown by the sample conductance values in Figure 5C, the increase in total leak caused by dendritic inhibition is 7.5 times larger (3 vs. 0.4) than the increase in dendritic leak caused by the same inhibitory conductance placed at the soma (see Table 1, lines 1–3).

**Table 1:** Total leak conductance (a.k.a. input conductance) and voltage attenuation in a passive 2-compartmental model.

(1) Total leak conductance *g_TotalL_*in the dendrite (node *d* in Figure 5c1) without inhibition	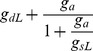	3.4
(2) Total leak conductance *g_TotalL_*in the dendrite (node *d* in Figure 5c2) with dendritic inhibition	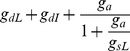	6.4
(3) Total leak conductance *g_TotalL_*in the dendrite (node *d* in Figure 5c3) with somatic inhibition	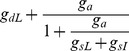	3.77
(4) Total leak conductanceat the soma (node *s* in Figure 4A & C) without inhibition	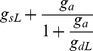	6.8
(5) Ratio of somatic to dendritic input conductance(*w/no inhibition*)	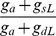	2
(6) Voltage attenuation from dendrite to somawith/no, or dendritic inhibition ( 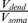 in Figure 4A, 5c2)	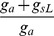	2.5
(7) Voltage attenuation from dendrite to somawith somatic inhibition ( 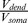 in Figure 4C, 5c3)	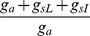	3.25

Definitions and example values: dendritic leak *g_dL_* = 1, dendritic inhibition *g_dI_* = 3, axial conductance *g_a_* = 4, somatic leak *g_sL_* = 6, and somatic inhibition *g_sI_* = 3. The following expressions were obtained by applying laws of electrical circuit analysis to nodes *d* and *s* in Figures 4 & 5 (viz. impedances in parallel & series, Kirchhoff's current law).

3. *Somatic inhibition steepens the attenuation of voltage signals, including NMDA spikes, as they travel passively from the dendrite to the soma. In contrast, dendritic inhibition co-localized with the excitation has no effect on the attenuation of voltage signals as they travel to the soma.*

Details. From 1 and 2 above, the dendritic compartment generates a constant-height NMDA spike regardless of the location or amount of inhibition. However, voltage attenuation from dendrite to soma depends on the circuit beginning with the axial conductance *g_a_* and moving rightward in Figs. 5C2 and 5C3. The divisive attenuation factor from dendrite to soma is therefore independent of the level of dendritic inhibition (Table 1, line 6), but can grow arbitrarily large as somatic inhibition is increased (Table 1, line 7). This divisive effect on spike height reiterates that seen in the compartmental model (Figure S8, see also [11]).

### The effect of dendritic inhibition depends systematically on its location relative to the excitation

#### Effects of distal vs. on-the-path inhibition: Experimental data

**Figure 6:**
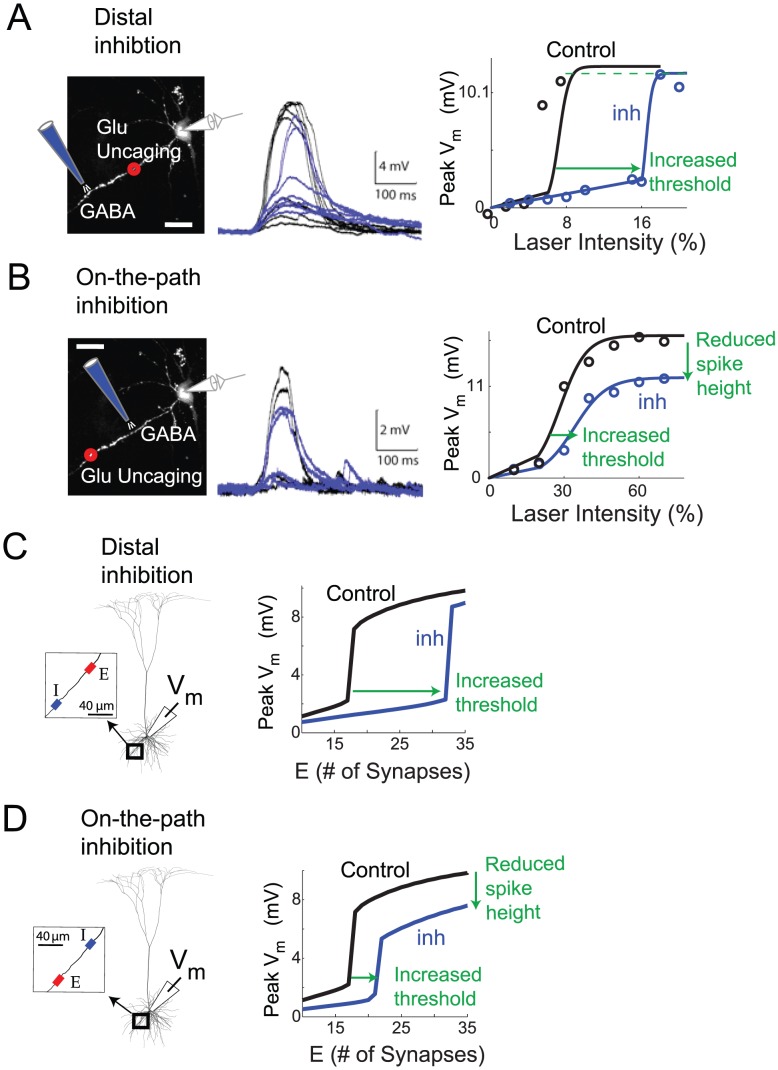
Effect of dendritic inhibition depends on location relative to excitation. (A,B) Voltage traces and i/o curves for whole-cell somatic recordings *in vitro*. In A, uncaging site was 75 µm from the soma while inhibition was 120 µm from the soma. Sites of excitation and inhibition were reversed in B. Excitation was delivered at least 10 ms after the iontophoresis. (C,D) I/O curves from the detailed compartmental model. Excitatory synapses (containing NMDA+AMPA conductance) were placed on a basal dendrite 125 µm from the soma. Inhibitory synapses (GABA_A_) were placed either 80 µm more distal than the excitation (C) or 80 µm more proximal, i.e. on-the-path to the soma (D). The red and blue rectangles in the C and D insets illustrate the spread of E and I types of synapses at their respective locations on the dendrite. The synapses were placed 0.5 µm apart as illustrated in Figure 1. Same number of GABA_A_- type synapses were activated in C,D. Each excitatory synapse in the simulations had 6 nS peak AMPA conductance. For the cases shown, peak inhibitory conductance was 20 nS.

Having established a clear dichotomy between somatic inhibition and dendritic inhibition co-localized with the excitatory stimulus, we carried out additional experiments to explore the effects of dendritic inhibition when the inhibition was either more distal than the excitation, or more proximal, that is, on the path to the soma. When inhibition was more distal than the site of glutamate uncaging (Figure 6A), the interaction closely resembled the co-localized case (see Figure 1B,F). Distal inhibition reduced the amplitude of the subthreshold EPSP by 48±7% (p = 0.006, paired Student's test, n = 4). However, despite this subthreshold shunting and the substantial increase in stimulus intensity needed to reach spike threshold (249±45%; p = 0.05, paired Student's test, n = 4,), NMDA spike height at the soma was again nearly unchanged (92±3% of control; p = 0.23, Student's t-test, n = 4).

**Figure 7:**
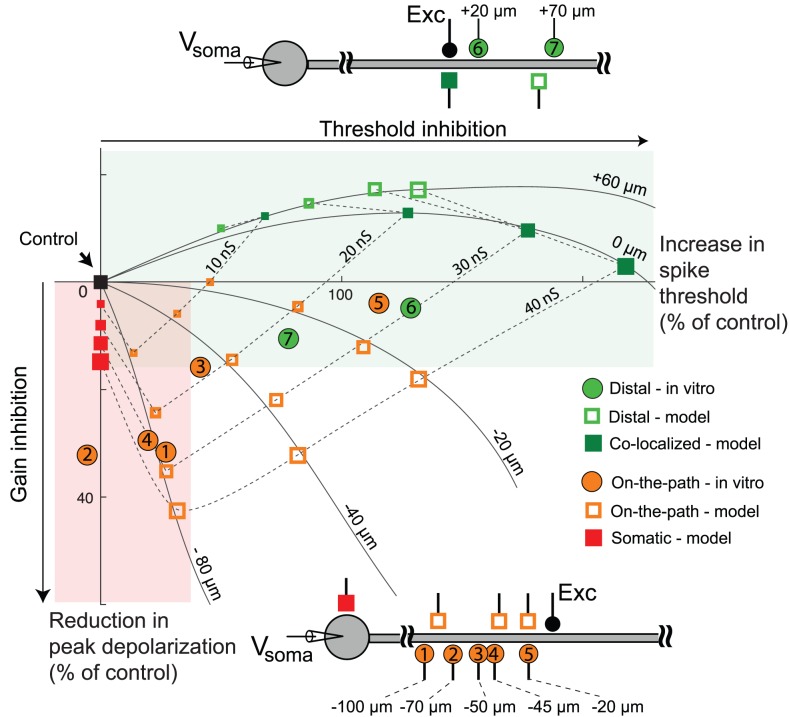
Summary of location-dependent effects of dendrite-targeting inhibition. This is expressed as joint % change in spike height and threshold relative to no-inhibition control (black square at origin). Red and green shaded areas were carried over from Figure 2 to indicate general trends for dendritic vs. somatic inhibition. *In vitro* data (red and green circles) were collected from different dendrites at different distal or on-the-path locations; separation distances between excitation and site of GABA iontophoresis are indicated in figure next to each data point. Results from detailed compartmental model are shown to provide context, including one representative location of more distal inhibition (open green squares) and three locations of on-the-path inhibition (open orange squares). Iso-inhibition and iso-location lines are splines fitted to the data points from the detailed compartmental model. Co-localized (filled green squares) and somatic (filled red squares) inhibition locations are shown for reference. In case of data points from the detailed compartmental model, size is indicative of strength. Simulations were carried out on an un-branched dendrite, though the results were similar for other dendrites.

In contrast, when inhibition was activated on the path between the excitation and the soma, we observed a mixture of somatic and dendritic effects (Figure 6B), that is, the inhibition significantly affected both the NMDA spike threshold and height. While it was not possible to make strict quantitative comparisons between model and data given that the data was collected from cells with different dendritic morphologies and excitation occurred at different distances from the soma, in both cases the relative amounts of gain vs. threshold inhibition depended systematically on the separation of on-the-path inhibition from the site of excitation (Figure 7, orange circles). Inhibition closer to the site of excitation mainly increased the threshold for NMDA spike generation (e.g. orange circle labeled −20 µm), whereas at larger separations, when inhibition moved closer to the soma, it mainly suppressed spike height (e.g. orange circle labeled −70 µm).

#### Effects of distal vs. on-the-path inhibition: detailed compartmental simulations

The detailed compartmental model replicated the experimental observations for “more distal” vs. “on-the-path” inhibition. As for the co-localized case, inhibition distal to the site of excitation raised the threshold but did not reduce spike height (Figure 6C), and in fact led to the same slight increase as in Figure 1E,F. In contrast, inhibition on the path to the soma led to a mixture of dendritic and somatic effects, that is, spike thresholds were elevated *and* spike heights were reduced (Figure 6D).

The effect of distal and on-the-path inhibition are summarized in Figure 7, showing a continuous morphing of the 2-dimensional pattern of threshold and height effects from the co-localized pattern (green squares horizontally distributed along the x-axis), to a sequence of on-the-path effects (three lines of orange open squares sweeping diagonally down), eventually approaching the somatic pattern (red squares, spread vertically down the y-axis). Further inspection of the distance-related effects revealed that:

1. The most effective location for increasing dendritic spike threshold is at the site of excitation. This is evident from the comparison of the effect of co-localized inhibition (solid green squares) with corresponding distal inhibition at +60 µm (open green squares) and on-path inhibition at −20 µm (open orange squares).
2. At equal distances from the excitatory stimulus, inhibition distal to the site of excitation is more effective than on-the-path inhibition at increasing the spike threshold (compare open green squares on the +60 µm arc to open orange squares on the −40 µm arc Figure 7).
3. The most effective location for suppressing dendritic spike height at the soma is on-the-path, and not at the soma (compare same strength inhibitory cases at various locations). This reiterates the finding of Koch et al. (1983) for inhibition of passive synaptic responses, and can be explained by slight elaborations of the electric circuit principles discussed above (see Discussion).

### Effect of GABA reversal potential

Given uncertainty about the net reversal potential of GABA-mediated inhibition *in vivo* [35]–[37] as well as in our experiments (Figure S1A), we modified the calculations relating to Figure 4 and carried out additional simulations in the detailed compartmental model to explore cases with the inhibitory reversal potentials ranging from −10 to +10 mV relative to the resting potential (Figure S5). The basic pattern of results with respect to the spike threshold and amplitude was unchanged, though with the elaboration that more negative reversal potentials exaggerate the threshold-increasing effects of inhibition in all cases, with little effect on spike height measured at the soma [38].

## Discussion

The very similar results of our experimental and modeling studies support the conclusion that dendritic and somatic inhibition exert very different effects on active dendritic integration in the thin perisomatic dendrites of pyramidal neurons. Dendritic inhibition, whether co-localized or more distal than the excitation, substantially increases the threshold level of excitation needed to trigger local spikes in the dendrites (in agreement with [13]), but does not effect spike height either locally or at the soma. In contrast, somatic inhibition slightly increases the threshold for dendritic spike generation, but has a divisive effect on the magnitude of dendritically generated spikes measured at the soma (Figure S8, Table 1, line 7). Finally, on-the-path dendritic inhibition modulates both the threshold and magnitude of the spike measured at the soma in a distance dependent manner. These location-dependent effects can also be characterized in terms of the changes inhibition causes in the branch's sigmoidal input-output curve measured at the soma: co-localized or more distal inhibition shifts the steep section of the sigmoid to the right by reducing the slope (and thus horizontally stretching) just the subthreshold portion of the input-output curve (Figure 1B,F, Figure 6A,C). In contrast, somatic inhibition mainly divides the amplitude of the entire sigmoid. Note that in the presence of somatic inhibition, the reduced asymptotic response at the soma for high levels of excitation does not imply a reduced peak voltage response at the site of dendritic spike initiation: we found that dendritic spike height was unaltered by inhibition whether applied directly at the site of spike generation or remotely at the soma.

Additionally, we found that beyond the obvious requirement that the excitation and inhibition must overlap in time in order to interact, the key location-dependent effects of inhibition we have explored depend little on the dynamics of dendritic spike generation per se: the 2-compartment model reproduces the basic effects of dendritic vs. somatic inhibition despite ignoring EPSP and IPSP time courses and all capacitive effects, and modeling synaptic and leak conductances only by their time-invariant I–V relationships. This implies that the different effects of dendritic vs. somatic inhibition on NMDA regenerativity arise primarily from the voltage-dependence of the NMDA channels rather than their kinetics [see also [38]]. Consistent with this lack of sensitivity to precise timing effects, the basic contrast between somatic and dendritic inhibition was preserved under various changes in timing parameters within physiological ranges (Figure S7). Simulations exploring inhibitory location effects under *in vivo*-like conditions show that the same pattern of results is maintained when inputs and outputs are measured in terms of average spike rates (see Figure S6, [39]).

### Mechanisms underlying inhibitory location effects

An analysis of the NMDA spike generation process in reduced electric circuit models explains the differential effect of dendritic vs. somatic inhibition (Figure 2B, 4 and S3). As a starting point, a graphical analysis of the NMDA and leak I–V curves in a single electrical compartment (Figure 5A,B, Text S1) predicts that the threshold number of NMDA channels needed to trigger a spike at a particular location should grow in proportion to the total input conductance at that location [see [30]]. Given this, inhibition anywhere in the cell, which causes a transient increase in the total leak conductance everywhere in the cell [12], should also produce an increase in the dendritic spike threshold everywhere in the cell. The location dependence of the effect depends on the degree to which a particular inhibitory input increases the leak conductance at a particular dendritic site, with the optimal location for “threshold inhibition” being directly at the site of spike generation. The potency of threshold inhibition falls off with distance from the site of excitation, but asymmetrically so: inhibition activated more distally than the site of excitation can produce substantial elevation of dendritic spike thresholds because the input resistance near the distal tip of a dendrite is originally high [32], [40] and is therefore particularly susceptible to lowering by the activation of additional membrane leak (see also [6]). (This assumes the site of distal inhibition is not so remote that its conductance-altering effects are negligible at the site of excitation – see Koch et al. 1983). Compared to distal inhibition, the threshold-elevating effect of on-the-path and somatic inhibition are much weaker: in addition to the increasing separation from the site of excitation as the inhibition moves towards the soma, the baseline input resistance drops substantially at more proximal sites due to larger branch diameters, the presence of branch points, and proximity to the soma itself [32], [40]. This leads to proportionally smaller increases in total membrane conductance when a given amount of inhibition is activated (Figure 7).

The location-dependent effect of inhibition on spike height measured at the soma is also asymmetric around the site of excitation. Inhibition co-localized with or distal to the site of excitation has no effect on spike height at the soma because it has no effect on the circuit that transmits the voltage signal from the site of excitation to the soma where the signal is measured. In contrast, inhibition on the path to the soma, or at the soma, increases the attenuation, and hence reduces the gain of voltage signals travelling from the dendrite to the soma by making the cable leakier ([11], Figure 6B,D, Figure 7). As in the case for passive conductances [11], the site of maximum effectiveness for inhibition of “response gain” at the soma lies neither at the site of excitation, nor at the soma, but at an intermediate point along the path (Figure 7).

### Relationship to other location-dependent effects of inhibition

The distinct effects of dendritic vs. somatic inhibition on dendritic spiking reported here extend the findings of Koch et al. [12] and Vu and Krasne [10] who studied inhibitory location effects in passive dendrites. Consistent with the theoretical predictions of Koch et al [12], [41], Vu and Krasne found that when excitatory conductances were weak, somatic and dendritic inhibition were largely interchangeable, in both cases divisively suppressing somatic voltage responses down to a fraction of their pre-inhibition values. This simple divisive effect was also seen in our experiments and modeling results for stimuli that remained subthreshold for NMDA spike generation (Figs. 1–4, 6).

In the suprathreshold range, we found Vu and Krasne's terms “relative” and “absolute” distinction can still be applied, but referring to different features of the excitatory response, and having a more complex relationship to the location of inhibition. In particular, Vu and Krasne called dendritic inhibition “relative” to imply that no matter how large a shunting inhibitory conductance is applied in the dendrite, its suppressive effect can always be overcome by a sufficiently strong excitatory conductance, which in the limit functions like a voltage clamp in the dendrite. In contrast, they termed proximal inhibition “absolute”, reflecting the fact that on-the-path or somatic inhibition *necessarily* lowers the asymptotic response that can be generated at the soma in the limit of strong dendritic excitation (see also [8]).

In active dendrites, the closest counterpart of relative inhibition is *relative threshold inhibition*, though the *relative* moniker is no longer uniquely tied to the dendrites: dendritic spikes are to varying degrees more difficult to trigger in the presence of inhibition at any location, whether co-localized, more distal, on-the-path, or somatic (see previous section). Once triggered by a sufficiently large excitatory stimulus, however, dendritic spikes are of full (pre-inhibition) height at the site of spike generation. In turn, the spiking dendrite counterpart of absolute inhibition is *absolute magnitude inhibition*, which includes not only somatic but on-the-path inhibition: any inhibition proximal to the site of spike generation increases the voltage attenuation that the dendritic spike experiences as it propagates to the soma, putting an absolute limit on the peak response that can be measured at the soma.

It is important to note that *relative* is not synonymous with *weak*, and *absolute* is not synonymous with *strong*. Inhibition placed at, or more distal, than the site of excitation, though *relative*, can under some circumstances have a stronger gain-suppressing effect measured at the soma than the same inhibitory conductance placed directly at the soma. For example, a dendritic inhibitory conductance that cuts the input resistance by a factor of two at the site of excitation, and thus cuts the subthreshold response at the soma by half, may have a negligible effect on the response at the soma when it is placed directly at the soma. Similarly, a dendritic inhibitory input is much better situated to veto dendritic spikes than an inhibitory input of the same size delivered to the soma [13].

Rhodes' [13] finding that somatic inhibition is ineffective at suppressing NMDA spikes arises from the fact that, unlike the relatively small inhibitory conductance needed to influence spike generation when activated at or near the dendritic site of spike generation, a much larger inhibitory conductance is needed at the soma to reduce spike height at the soma, given the already low input resistance at the soma. A similar effect likely accounts for the relatively greater suppression of excitatory responses by dendritic compared to somatic inhibition in a recent experimental study [29]. Large inhibitory conductances have in fact been measured at the soma in intracellular recordings both *in vitro* and *in vivo* [42]–[45].

### Additional effects of inhibition on spike height

Though it is a straightforward outcome of our time-invariant 2-compartment model, the fact that synaptically evoked NMDA spikes are essentially unchanged in height even when powerful inhibitory conductances are activated directly at the site of spike generation seems surprising in the context of classical synaptic integration effects. In particular, inhibition is generally expected to reduce the magnitude of an excitatory synaptic response in a graded fashion, especially when the excitatory and inhibitory synapses are co-localized. The all-or-none character of NMDA spikes in the presence of inhibition seen both in our models and our electrophysiological data is less surprising, however, when it is recalled that conventional fast action potentials are also stereotyped in height despite orders-of-magnitude differences in input resistance both within (axon vs. soma) and between (small and large) cells. Our observations here support the conclusion that NMDA currents, like other types of spiking mechanisms, produce relatively stereotyped responses once they are driven into the regenerative range, despite the substantial differences in input resistance found in different cellular locales at different moments in time.

Interestingly, this principle of invariant spike height was violated in one of our results: we found that our detailed compartmental model produced modest *increases* in spike height in the presence of strong dendritic inhibition (Figure 1F). This effect fell outside the scope of our time-invariant 2-compartment analysis, and was observed only in the presence of a relatively fast-decaying inhibitory conductance in our detailed compartmental model (Figure S7). When NMDA and inhibitory conductances both remain near their peaks for longer times, NMDA spike height is determined by the balance point between the inward (NMDA) and outward (leak+inhibition) membrane currents, as indicated by the intersection of the red and green I–V curves in Figure S4. If the inhibitory conductance decays from its peak more rapidly than the NMDA conductance, the slope of the green “total leak” I–V curve begins to decrease in mid response, causing the balance point to slide to the right on the I–V graph, which in turn leads to an increase in spike height. In this light, the lack of an increase in spike height on average for dendritic inhibition in our electrophysiological data was most likely explained by the slow decay time of the inhibition delivered by GABA iontophoresis. Whether rapidly or slowly decaying inhibition is the better model for an intact cortical circuit depends on the situation: a relatively fast inhibition may be more physiologically relevant for discrete stimulation, such as a brief perturbation of a whisker, whereas inhibition impinging on a neuron in the form of sustained high frequency trains may be better modeled as tonic inhibition. It is worth noting, however, that for either brief or long lasting inhibition relative to the NMDA activation, we found that dendritic inhibition was always associated with the elevation of dendritic spike thresholds (Figure S7), and was never associated with reductions in dendritic spike height.

### A spectrum of inhibitory effects: flexibility for cortical circuits

The distinction between threshold and gain inhibition depending on the location of the inhibitory synapses suggests an anatomical scheme that cortical circuits could use to tailor their local circuit computations. Depending on the degree to which a particular axon pathway is supposed to exert threshold vs. gain inhibition on its PN targets, that pathway would, in appropriate amounts, drive dendrite vs. soma-targeting interneurons in the vicinity. The same rule could apply to inhibitory interneurons that target other inhibitory interneurons: those wanting to *relieve* PNs of gain suppression might inhibit soma-targeting interneurons, while those wanting to lower spike thresholds in PN dendrites would target dendrite-targeting interneurons. It is also possible that inhibitory interneurons are themselves subject to the location effects reported here for PNs. This seems plausible in light of a recent report that interneurons in the CA1 region of the hippocampus produce dendritic spikes similar to those seen in pyramidal neurons [46].

#### When location of inhibition may not matter

In considering whether the present results can be extrapolated to the more complex situation where multiple branches or the entire dendritic tree is stimulated, caution is in order since the outcome is likely to depend heavily on the spatial pattern of excitation that drives the dendritic tree. A key point is that in cases where excitation remains in the “linear” range, i.e. subthreshold for local spike generation or other nonlinear effects, dendritic and somatic inhibition are nearly indistinguishable. Both produce graded, divisive suppression of response magnitudes at the soma (Figures 1B,D,F,H). The same conclusion can be drawn from the passive data and modeling results of Koch et al. [12] and Vu and Krasne [10]. One implication of this is that, if pyramidal neurons are routinely driven by diffuse patterns of excitation *in vivo*, where many dendrites are weakly stimulated and thus remain within their linear ranges [47], the functional distinction between somatic and dendritic inhibition would be reduced or eliminated – compare the subthreshold ranges of Figures 1B, D, F, H. Thus, rather than suggest answers, the “diffuse stimulation” hypothesis leaves open the question as to why interneurons in the cortex do in fact specifically target somatic, dendritic, and other subdomains of PNs. By contrast, in the scenario in which PN dendrites routinely receive spatio-temporally concentrated inputs that drive their dendrites to spike [34], [48], [49]), they are subject to a rich spectrum of gain and threshold suppression effects by the cortical and subcortical pathways that drive and modulate their responses.

#### Similarity of excitatory and inhibitory location effects

The location-dependent inhibitory effects reported here are intriguingly similar in form, though opposite in direction, to location-dependent excitatory modulation effects we have recently described in these same dendrites [50]. In that related study, we found that excitatory inputs to PN basal dendrites also differently affect a dendrite's sigmoidal input-output curve depending on their location: a distal excitatory input lowers the threshold for an NMDA spike triggered by a more proximal input, that is, it left-shifts the proximal input's sigmoidal i/o curve. In contrast, a proximal input both lowers the threshold and increases the gain of the sigmoidal response to a more distal input, analogous to the combined threshold and gain effects associated with on-the-path inhibition. The very similar form of these excitatory and inhibitory modulation effects strengthens the case that PN thin dendrites, by virtue of their voltage-dependent NMDA currents and asymmetric cable properties, possess significant nonlinear analog processing capabilities tied to synapse location [51], [52] These include the ability for excitatory and inhibitory modulatory pathways to bi-directionally manipulate the thresholds and gains of dendritic i-o curves through biases in the spatial distribution of their synaptic influences along the proximal-distal axis of perisomatic thin dendrites. In the case of excitation, biases would be established in the direct excitatory projections onto PN dendrites. In the case of inhibition, biases would be established indirectly by manipulating the relative activation of dendrite- vs. soma-targeting interneurons.

This view that the neocortex can achieve graded, bidirectional modulation of dendritic input-output curves through spatial biasing of excitatory and inhibitory influences along the proximal-distal axis of PN thin dendrites represents a significant departure from our conceptual starting point, the “2-layer model” of the pyramidal neuron [28], [34], [53]–[55]. According to that simpler model, the response of a dendrite depends on the strengths of its synaptic inputs but not their locations. Analog location effects within individual dendrites [12], [40], [49], [51], [56] open up the potential for a much wider range of local circuit computations within the same compact physical hardware.

Additional experimental and modeling studies will be needed (1) to describe the effects of inhibition targeted to other parts of the cell (including the apical tuft and the axon initial segment), (2) to determine whether excitatory and inhibitory location effects combine in predictable ways when they occur together, and (3) to assess the degree to which location effects generalize across neuron morphological types.

## Methods

### Ethics statement

All experimental procedures were in accordance with guidelines of the Technion Institutional Animal Care and Use Committee.

### Electrophysiology

Cortical brain slices were prepared from the somatosensory cortex from 20–40 day old male Wistar rats. All experimental procedures were in accordance with guidelines of the Technion Institutional Animal Care and Use Committee. The neurons were visualized using a confocal scanning microscope (Olympus 1000) equipped with infrared illumination and Dot contrast optics combined with infrared video enhanced microscopy [32]. Whole-cell patch-clamp recordings were made from visually identified layer 5 pyramidal neurons using infrared– differential interference contrast optics. The extracellular solution contained the following (in mM): 125 NaCl, 25 NaHCO3, 25 glucose, 3 KCl, 1.25 NaH2PO4, 2 CaCl2, and 1 MgCl2, pH 7.4 (at 35–36°C). The intracellular solution contained the following (in mM): 115 K-gluconate, 20 KCl, 2 Mg-ATP, 2 Na2-ATP, 10 Na2-phosphocreatine, 0.3 GTP, 10 HEPES, and 0.2 Oregon Green 488 Bapta-1 (OGB-1), pH 7.2. The electrophysiological recordings were performed using Multi-Clamp 700A (Molecular Devices, Foster City, CA), and the data were acquired and analyzed using pClamp 8.2 (Molecular Devices), a homemade software, and Igor (Wavemetrics, Lake Oswego, OR) software. Statistical tests were performed using Excel software (Microsoft, Redmond, WA).

Full images were obtained with a temporal resolution of 1 Hz, and in the line scan mode with a temporal resolution of 512 Hz. Images were analyzed using Tiempo (Olympus), homemade software, and Igor software. Fluorescence changes were quantified as increase in fluorescence from baseline normalized by the baseline fluorescence (ΔF/F as a percentage). The background florescence was subtracted from all measurements before calculation of the ΔF/F. Calcium transients are reported as mean± SD.

UV laser glutamate uncaging was used to deliver excitation in all except two experiments for somatic inhibition, in which case electrical stimulation was used. For the uncaging experiments, caged glutamate [4-methoxy-7-nitroindolinyl (MNI)-glutamate; Tocris, San Diego, CA] was photolyzed with a 361 nm UV-laser beam (Enterprise 2; Coherent, Palo Alto, CA) using point scan mode. The caged glutamate (5–10 mM) was delivered locally to a branch using pressure ejection (5–10 mbar) from an MNI-glutamate-containing electrode (2 micron diameter). Uncaging spots were selected on dendrites that did not have neighboring branches both in the XY plane and above or below them. Focal synaptic stimulation was performed with a theta patch pipette (3–10 MΩ resistance) located in close proximity (2–5 microns) to the selected basal dendritic segment. Stimulation duration was 0.1 ms, in a constant voltage mode. GABA was delivered by way of iontophoresis through a pipette (6–15 MΩ resistance; 500 mM) positioned adjacent to the cellular membrane. The effect of iontophoresis sensitively depended on the distance between the electrode to the cell, with about 2 fold decrease in IPSP amplitude with distances larger than 2 microns [57]. The iontophoresis intensity was 2–4 nA (pulse width-2 ms), unless stated otherwise. The stimulating electrodes were filled with Alexa Fluor 633 to position them in accordance with the fluorescent image of the dendrite.

#### I/O Curves

The peak membrane depolarization for a given level of excitatory stimulation (open circles in Figure 1B,D and Figure 6C,D) was used to plot the i/o curves (solid lines in Figure 1B,D and Figure 6C,D). The curves were obtained with best fits of piece-wise linear and sigmoidal function to the data. Data points below the spike threshold (determined as the stimulation strength resulting in the maximum change in EPSP in the raw data) were fitted with a line through the origin and the data point just below threshold. All data points above threshold (including the threshold point) were fitted with a sigmoid. The specific sigmoidal function used was a logistic function as described below:
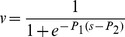
Here, 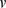 and 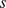 were the membrane depolarization and the stimulation strength from the *in vitro* data set, while 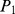 and 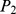 were the fitting parameters.

### The detailed compartmental model

To confirm the predictions of the 2-compartment model regarding the location of inhibition, we used a detailed compartmental model of a reconstructed layer-5 pyramidal neuron [58], [59]. The passive cable properties, voltage-dependent Na+ and K+ channel densities and NMDA-to-AMPA peak conductance ratio (Table 2) were derived from *in vitro* electrophysiological recordings in Layer-5 pyramidal cells [32]. The GABA_A_-type inhibitory conductance was based on the model of [31]. Excitatory synapses were placed 0.5 µm apart about 125 µm from the soma unless otherwise stated. In cases where dendritic inhibition was modeled, the inhibitory synapses were either: a) co-localized with the excitation, b) more distal than the excitation, or c) on the path to the soma. The single pulse stimulus was 0.1 ms in duration. When spike train stimuli were used (Figure S6), both excitatory and inhibitory synapses were driven by independent 50 Hz Poisson trains.

**Table 2:** Multi-compartment model parameters.

Property		Value	Details
**Passive Properties**	R_m_	*dendrite*: 10 KΩ.cm^2^	
		*node*: 50 Ω.cm^2^	
		*other*: 20 KΩ.cm^2^	
	C_m_	*myelination*: 0.05 µF/cm^2^	
		*soma*: 1 µF/cm^2^	
		*dendrites*: 2 µF/cm^2^	
	R_a_	100 Ωm	
**Active Properties**	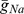	*soma*: 25 pS/µm^2^	[32]
		*dendrites* ( 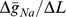 ) : 0.003 pS/µm^3^	
		*axon IS, hillock* and *nodes*: 100 pS/µm^2^	
		*myelination*: 0.6 pS/µm^2^	
	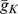	*soma*: 3 pS/µm^2^	
		*dendrites*: 0.03 pS/µm^2^	
		*axon IS*, *hillock* and *nodes*: 5 pS/µm^2^	
		*myelination*: 200 pS/µm^2^	
**Synaptic Conductances**	AMPA	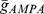 = 1.5 nS	[54]
		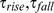 = 0.05, 0.5 ms	
	NMDA	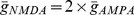	
		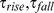 = 2.1, 18.8 ms	
		**2-state kinetic model**	[63]
		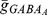 as stated in Figures 1, 2, 5, 6	
	GABA_A_	**Biexponential model** (Figure S7)	
		Short: τ_1_ = .5 ms, τ_2_ = 100 ms	
		Long: τ_1_ = .5 ms, τ_2_ = 2 ms	

### The reduced model

#### NMDA channel model

This study concerns the effects of inhibition on NMDA spike generation. The NMDA channel behavior and the methods we used to study NMDA spike threshold and magnitude are shown in Figure S3. A single-compartment circuit is shown in Figure S3A containing a passive leak conductance with reversal potential *E_L_* = −70 mV and an NMDA conductance with a reversal potential of 0 mV, typical for an excitatory synapse. We used a standard model for the NMDA conductance (adapted from [30]):
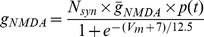
(1)with,
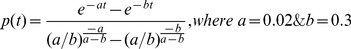
representing the conductance time course (independent of Mg^++^ block) as a result of different binding and unbinding kinetics of NMDA channels [60], and the voltage-dependent denominator term representing the Mg^++^ block that suppresses most current flow at negatively polarized potentials. The strength of excitation was controlled by the variable *N_syn_*, representing the number of glutamate activated NMDA channels, with constant 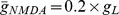, denoting the unblocked single channel conductance. A plot of the numerator of equation 1 is shown in Figure S3B for increasing values of *N_syn_*; three colored cases (*N_syn_* = 15, 21, 25) are carried through Figure S3 to illustrate NMDA response just below (orange) and above (red, burgundy) the NMDA spike threshold. Asterisks indicate times at which *p(t)* is at its maximum value of 1. Capacitive currents were ignored based on the assumption that the NMDA conductance remains at or near its peak value long enough for the membrane to reach its equilibrium potential — an imperfect but reasonable assumption given existing data from pyramidal neurons [24], [30]. This assumption was further corroborated by the fact that the 2-compartment version of this model confirmed our findings in slice preparation as well as a detailed compartmental model.

#### Computing *V_m_*

The voltage-dependence of the NMDA channel prevented an algebraic solution of the circuit shown in Figure S3A, so we computed *V_m_(t)* numerically from the circuit equation given by Kirchhoff's current law:
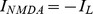


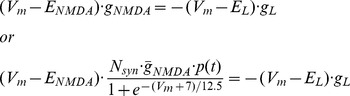
(2)If multiple values of *V_m_* satisfied equation 2, we used the value closest to the resting potential [61].

Explicit traces of the time-varying membrane potentials, that is, the NMDA EPSPs, are shown in Figure S3D for a range of excitation strengths. Orange and red curves show responses that are subthreshold and suprathreshold for NMDA spike generation, respectively. The burgundy curve illustrates the saturation of NMDA spike height once the threshold has been crossed. Voltage traces in Figure S3D and the sigmoidal pattern of voltage peaks shown in Figure S3E are both typical of responses of thin dendrites of neocortical pyramidal cells activated with increasing stimulus intensities [24], [30], [62].

The mechanism of NMDA-based non-linearity (Figure S3D,E) is illustrated graphically (Figure S3C) via I–V plots for NMDA and leak currents on the left and right sides of equation 2, respectively: the green line represents the leak current as a function of voltage (from Ohm's law), drawn reflected below the x-axis due to its leading negative sign. The non-monotonic grey and colored curves represent the instantaneous NMDA I–V relations for different values of *N_syn_.p(t)* at different moments in time. Colored dashed arrows suggest the temporal evolution of an NMDA I–V curve as *p(t)* increases and decreases through time (shown explicitly in Figure S3B). Each colored I–V curve shows the instantaneous I–V relationship that exists at the moment of peak NMDA conductance (when *p*(*t*) = 1) for one of the three levels of NMDA excitation highlighted in Figure S3B.

The solution *V_m_(t)* to equation 2 at any given time corresponds graphically to the intersection between the *I_NMDA_* and −*I_L_* curves. These intersection points give the voltages at which the inward (NMDA) and outward (leak) currents are balanced at a stable equilibrium. As the NMDA conductance waxes and wanes through time (see dashed arrows), the equilibrium point slides back and forth along the green line, tracing the progress of the membrane potential in response to NMDA excitation. Colored dots indicate the peak voltages reached for the three levels of excitation highlighted in Figure S3B.

#### Identifying NMDA spike threshold and height on I–V plots

When the excitation level was subthreshold for NMDA spike generation (e.g. orange case), the balance point at *t_peak_* (orange dot) lay at a deeply polarized potential close to rest on the I–V plot in Figure S3C; this explains the small amplitude of the orange EPSP in Figure S3D. The spike threshold is the lowest level of excitation *N* at which the descending slope of the NMDA I–V curve skims below the green line to run entirely within the grey shaded region. When this occurs (red I–V curve), the balance point jumps to a far more depolarized value (red dot); this value corresponds to the height of the NMDA spike at threshold (black dashed line). Once past the spike threshold, further increases in NMDA excitation (burgundy case) produced only marginal increases in spike height (burgundy dot).

#### The 2-compartment model

A 2-compartment model was used to study the effects of dendritic vs. somatic inhibition on NMDA spike threshold and height as seen at the soma (Figure 4A,B). The dendritic compartment contained NMDA conductance and a leak conductance *g_dL_* as in Figure S3A, and an inhibitory conductance *g_dI_* in cases involving dendritic inhibition. The reversal potential of the inhibitory conductance was equal to the resting potential *E_I_* = *E_L_* = −70 mV. The somatic compartment contained a leak conductance *g_sL_* and an inhibitory conductance *g_sL_* in cases involving somatic inhibition. The two compartments were coupled by an axial conductance *g_a_*, which was adjusted to achieve passive voltage attenuation between the dendritic and somatic compartment of at least a factor of 5 (depending on the level of somatic inhibition). As in the single-compartment model of Figure S3, capacitive currents were ignored. Steady state voltage responses and NMDA spike threshold and height were calculated using the methods described above, but with the modified KCL equation
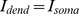
where,
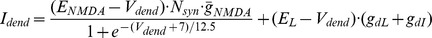
and
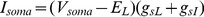
(3)and exploiting the relationship between *V_soma_* and *V_dend_*:
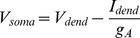
(4)to eliminate the dependence on *V_soma_* in equation 3. The resulting equation was solved numerically for *V_dend_*, then *V_soma_* was computed from equation 4.

The I–V curves from the perspective of the soma illustrate the differential effect of dendritic (Figure S4A) vs. somatic (Figure S4B) inhibition on the magnitude of the NMDA spike.

## Supporting Information

Figure S1 Characterizing the effect of GABA iontophoresis in slice preparation. (**A**) Examples of IPSPs evoked by GABA iontophoresis at the soma. IPSPs evoked by GABA iontophoresis at the soma had only a fast component in 12 of the cells, with an average (±SD) amplitude of −0.65±4.5 mV at rest, rise time of 33.5±19.7 ms and decay time of 158.6±89.9 ms. In 10 cells the somatic IPSP had a slower component with amplitude of −1.74±3.22 mV, rise time of 172.4±231.1 ms and decay time of 558.6±242.3 ms. 3 cells responded with both slow and fast components. Dendritic IPSPs were smaller and on average faster; in 9 cells the dendritic IPSP had a fast component with amplitude of 0.96±1.31 mV, rise time of 35.6±17.1 ms and decay time of 101.7±58.4 ms. Two cells responded with additional slow component with amplitude of 1.5±0.7 mV, rise time of 151.5±111 ms and decay time of 550±353.5 ms. (**B**) The effect of somatic inhibition on input resistance was intensity dependent: at control, the recorded input resistance of the neuron was 61.7±12.6 MOhm. GABA iontophoresis at a current intensity of 3 nA reduced the input resistance by about half to 34.5±11.7 MOhm and at 5 nA to 14.6±5.03 MOhm. (**C**) Representative somatic EPSP recording without (red) and with GABA iontophoresis (black). The excitatory activation (uncaging in this case) was done 200 µm from the soma and was delayed with respect to the iontophoresis. The site of iontophoresis was 100 µm from the soma. Notice the delayed onset of excitation with respect to iontophoresis. (**D**) Overlayed traces for 4 different values (100, 200, 1000 & 2000 ms) of delayed onset of excitation for the experiment in (**C**). The suppression of maximal NMDA spike amplitude depended on the onset delay.
(PDF)

Figure S2 Experiments in the detailed compartmental model to measure input resistance changes at the somatic and dendritic location of inhibition. Inhibitory conductances of increasing strength were activated under current clamp at the soma and the dendritic location. The peak input resistance was measured as the ratio of membrane potential trough and the clamp current. Note that both the X and Y-axes for the input resistance graphs on the bottom are dissimilar.
(PDF)

Figure S3 Model of NMDA channel and definitions of NMDA spike threshold and height. (A) A single compartment model of neural membrane with an NMDA and leak conductance. (B) Example time courses of peak NMDA conductance for different values of N_syn_. Asterisk indicates time at which p(t) = 1. (C) I–V curves for leak (I_L_) and steady-state (i.e. p(t) = 1) NMDA conductance (I_NMDA_) in the single compartment. Mathematical formulation of NMDA conductance shows a dependence on both membrane voltage and time-dynamics [54]. Also shown are the equations for steady-state value of V_m_. The I–V plots show the resulting V_m_ (colored dots) for the different values of N_syn_ shown in B: it is the voltage for which net inward current (I_NMDA_) is balanced by the outward current (I_L_). These I–V plots can also give us an idea of how the membrane voltage will change as p(t) changes in time, as shown in B. They can be thought of as instantaneous I–V curves giving an estimate for V_m_ (as described above) for different values of N_syn_×p(t). The “Time” arrow follows the I–V curves for I_NMDA_ as N_syn_×p(t) changes in time. The intersection of each of these time-changing I–V curves with the leak I–V curve (I_L_, reflected in green) gives an estimate for V_m_ as a function of time. Notice that as N_syn_×p(t) changes follow the yellow curve in B, the intersection of resulting I–V curves with the leak I–V curve is a linear progression similar to the linear rise and fall of V_m_ in D (yellow curve). When N_syn_×p(t) changes follow the red curve in B, the intersection of resulting I–V curves with the leak I–V curve involves a non-linear jump (yellow dot and red dot) leading to the non-linear rise and fall of V_m_ in D (red curve). Any value of N_syn_ larger than this will ensure a non-linear jump in the I–V domain (all curves below red) and consequently the time domain (D, all curves above red). Thus, spikes in the time domain correspond to all the NMDA I–V curves in C whose negative slope region lies completely below the -I_L_ (green) l-V curve, as shown. Specifically, the smallest N_syn_ (red curve) for which this is the case is a representative of the minimum conductance required, given the membrane leak, to generate a spike in time-domain. The exact N_syn_ would depend on the exact time-course of the conductance and the membrane capacitance. (D) V_m_ at the single compartment in the presence of time-varying NMDA conductance, as described in C. Sufficient value of N_syn_ (red) leads to a non-linear jump in peak V_m_ - the NMDA spike. (E). I/O curve for peak values of voltage traces shown in D. Notice the NMDA-based super-linear jump in the function.
(PDF)

Figure S4 I–V curves at the soma in a 2-compartment model. Line colors are the same as in Figure 5B. (A) Somatic I–V curves in case of dendritic inhibition. Analysis of spike threshold and height is similar to that of Figures 5 and S3, except voltage attenuation from dendritic compartment where NMDA spike is generated, to somatic compartment where voltages are measured, leads to same horizontal compression of both NMDA (red) I–V curves. In particular, steep negative slope sections of NMDA I–V curves, and corresponding spike thresholds (red dots) are pushed proportionally closer to rest, reflecting the attenuated view of the dendritic NMDA spike at the soma (same attenuation with and without inhibition). (B) Somatic I–V curves in case of somatic inhibition. Consistent scaling of NMDA and leak I–V curves, as occurs in the dendritic compartment (Figure 4A), is disrupted from the perspective of the somatic compartment: total leak conductances scales with increasing somatic inhibition (green line), but NMDA I–V curve does not because voltage attenuation (and corresponding horizontal compression of the NMDA I–V curve) is greater with than without somatic inhibition.
(PDF)

Figure S5 Effect of reversal potential of the inhibitory conductance on the location effect. I/O curves for somatic inhibition (A,B) and dendritic inhibition (C,D) for 3 levels of inhibitory reversal potential: −60 mV (dashed), −70 mV(solid), −80 mV (dotted). The resting membrane potential was −70 mV in both the 2-compartment (A,C) and the detailed compartmental model (B,D).
(PDF)

Figure S6 A conceptual model for location effect of inhibition in the presence of dendritic NMDA spikes. Assuming firing rates are determined as an average of synaptic activity over a few hundred milliseconds, this can be modeled as an averaging/pooling operation (B) over the single pulse subthreshold response (A). Coupled with a threshold-linear function for the somatic spiking mechanism, we get the predictions for suprathreshold i/o curves for both somatic and dendritic location of inhibition (C). (D) shows firing rates as a function of excitatory synapses based on simulations in a detailed compartmental model. The inhibitory synapses were either co-localized with the dendritic excitation or placed at the soma.
(PDF)

Figure S7 Sensitivity of the location effect to the onset and duration of inhibition. (A) Representative location of excitatory (red square) and inhibitory (blue/dark green/light green circle) synapses in the morphologically detailed model. (B) I/O function between peak membrane potential and strength of excitation for the case of no inhibition (black), slow (blue), fast (dark green), and delayed fast (light green) inhibition in a detailed compartmental model. For the cases shown here, the peak inhibitory conductance was 4 nS for dendritic inhibition and 24 nS for somatic inhibition. (C) Summary of the effect of inhibition offset and duration at the somatic (triangles) and dendritic location (circles) in the detailed model, expressed as joint % change in spike height and threshold relative to no-inhibition control (black square at the origin). Peak conductance for dendritic inhibition cases shown here was 4, 8, 12, 16 and 20 nS, while that for somatic inhibition was 24, 48, 72, 96 and 120 nS. Each excitatory synapse in this experiment had 6 nS peak AMPA conductance. Excitatory synapses with 1.5 nS peak AMPA conductance with similar distribution of density along the dendrite gave similar results. Red and green shaded areas were carried over from Figure 2 to indicate general trends for dendritic vs. somatic inhibition in the *in vitro* data.
(PDF)

Figure S8 Comparison of divisive (green) and subtractive (blue) prediction for the computational effect of somatic inhibition on somatic membrane potential in the detailed model. Red squares are data points from simulation of the detailed compartmental model (also shown in Figure 2). The asterisk indicates the data point used for generating the prediction.
(PDF)

Text S1 Explanation for constancy of NMDA spike height with I–V plots.
(PDF)
