# Supplementary material for: Location-Dependent Effects of Inhibition on Local Spiking in Pyramidal Neuron Dendrites
Source: PLoS Comput Biol. 2012 Jun 14;8(6):e1002550. doi: 10.1371/journal.pcbi.1002550 (PMC3375251; doi:10.1371/journal.pcbi.1002550)
Supplement: Figure S1 — Characterizing the effect of GABA iontophoresis in slice preparation. (A) Examples of IPSPs evoked by GABA iontophoresis at the soma. IPSPs evoked by GABA iontophoresis at the soma had only a fast component in 12 of the cells, with an average (±SD) amplitude of −0.65±4.5 mV at rest, rise time of 33.5±19.7 ms and decay time of 158.6±89.9 ms. In 10 cells the somatic IPSP had a slower component with amplitude of −1.74±3.22 mV, rise time of 172.4±231.1 ms and decay time of 558.6±242.3 ms. 3 cells responded with both slow and fast components. Dendritic IPSPs were smaller and on average faster; in 9 cells the dendritic IPSP had a fast component with amplitude of 0.96±1.31 mV, rise time of 35.6±17.1 ms and decay time of 101.7±58.4 ms. Two cells responded with additional slow component with amplitude of 1.5±0.7 mV, rise time of 151.5±111 ms and decay time of 550±353.5 ms. (B) The effect of somatic inhibition on input resistance was intensity dependent: at control, the recorded input resistance of the neuron was 61.7±12.6 MOhm. GABA iontophoresis at a current intensity of 3 nA reduced the input resistance by about half to 34.5±11.7 MOhm and at 5 nA to 14.6±5.03 MOhm. (C) Representative somatic EPSP recording without (red) and with GABA iontophoresis (black). The excitatory activation (uncaging in this case) was done 200 µm from the soma and was delayed with respect to the iontophoresis. The site of iontophoresis was 100 µm from the soma. Notice the delayed onset of excitation with respect to iontophoresis. (D) Overlayed traces for 4 different values (100, 200, 1000 & 2000 ms) of delayed onset of excitation for the experiment in (C). The suppression of maximal NMDA spike amplitude depended on the onset delay. (PDF) [file pcbi.1002550.s001.pdf]

Figure S1, related to figures 1, 5, 6

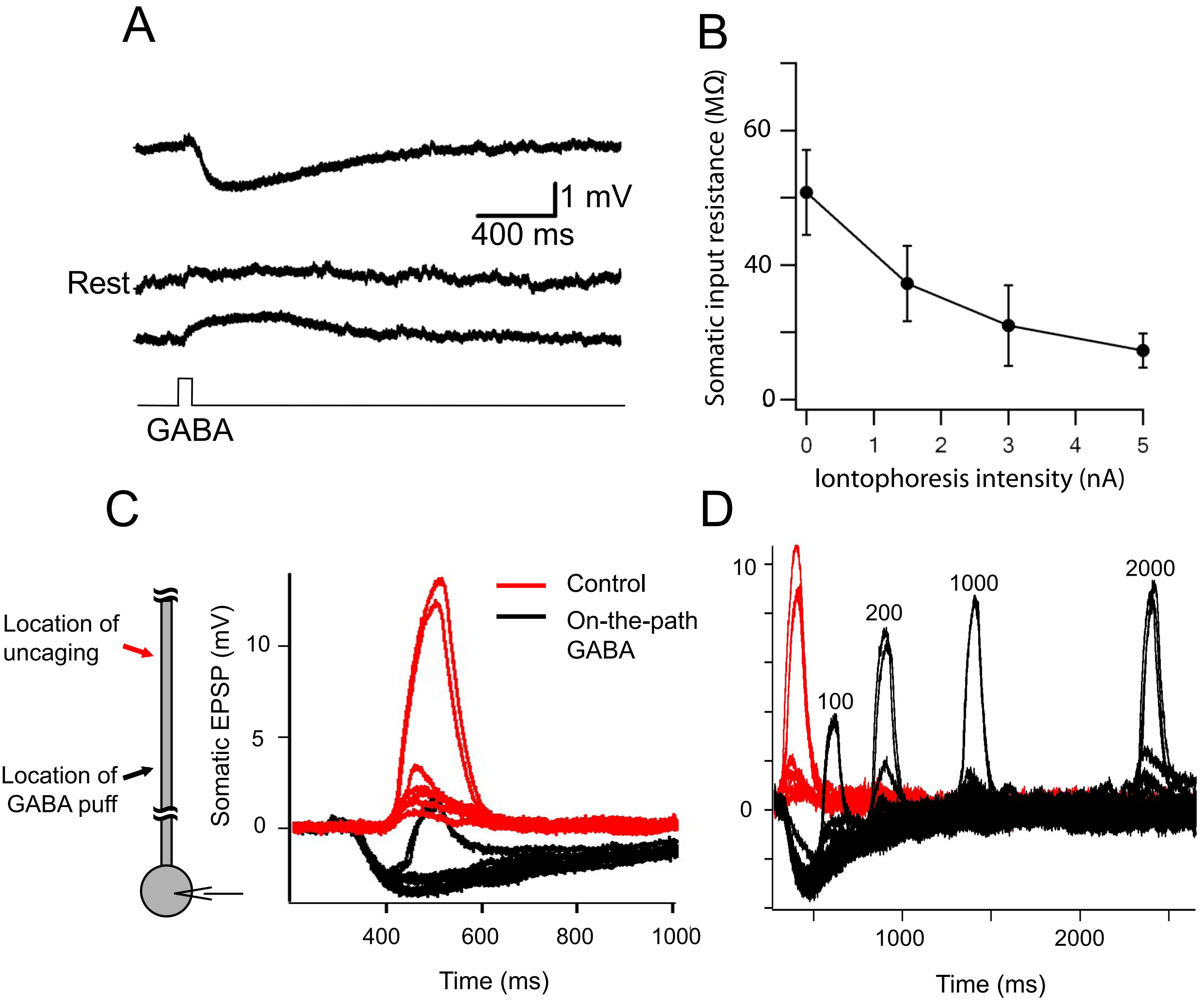

Figure S1 Characterizing the effect of GABA iontophoresis in slice preparation. (A) Examples of IPSPs evoked by GABA iontophoresis at the soma. IPSPs evoked by GABA iontophoresis at the soma had only a fast component in 12 of the cells, with an average (±SD) amplitude of -0.65±4.5 mV at rest, rise time of 33.5±19.7 ms and decay time of 158.6±89.9 ms. In 10 cells the somatic IPSP had a slower component with amplitude of -1.74±3.22 mV, rise time of 172.4±231.1 ms and decay time of 558.6±242.3 ms. 3 cells responded with both slow and fast components. Dendritic IPSPs were smaller and on average faster; in 9 cells the dendritic IPSP had a fast component with amplitude of 0.96±1.31 mV, rise time of 35.6±17.1 ms and decay time of 101.7±58.4 ms. Two cells responded with additional slow component with amplitude of 1.5±0.7 mV, rise time of 151.5±111 ms and decay time of 550±353.5 ms. (B) The effect of somatic inhibition on input resistance was intensity dependent: at control, the recorded input resistance of the neuron was 61.7±12.6 MOhm. GABA iontophoresis at a current intensity of 3 nA reduced the input resistance by about half to 34.5±11.7 MOhm and at 5 nA to 14.6±5.03 MOhm. (C) Representative somatic EPSP recording without (red) and with GABA iontophoresis (black). The excitatory activation (uncaging in this case) was done 200 μm from the soma and was delayed with respect to the iontophoresis. The site of iontophoresis was 100 μm from the soma. Notice the delayed onset of excitation with respect to iontophoresis. (D) Overlayed traces for 4 different values (100, 200, 1000 & 2000 ms) of delayed onset of excitation for the experiment in (C). The suppression of maximal NMDA spike amplitude depended on the onset delay.
