# Supplementary material for: Location-Dependent Effects of Inhibition on Local Spiking in Pyramidal Neuron Dendrites
Source: PLoS Comput Biol. 2012 Jun 14;8(6):e1002550. doi: 10.1371/journal.pcbi.1002550 (PMC3375251; doi:10.1371/journal.pcbi.1002550)
Supplement: Figure S2 — Experiments in the detailed compartmental model to measure input resistance changes at the somatic and dendritic location of inhibition. Inhibitory conductances of increasing strength were activated under current clamp at the soma and the dendritic location. The peak input resistance was measured as the ratio of membrane potential trough and the clamp current. Note that both the X and Y-axes for the input resistance graphs on the bottom are dissimilar. (PDF) [file pcbi.1002550.s002.pdf]

Figure S2 Jadi

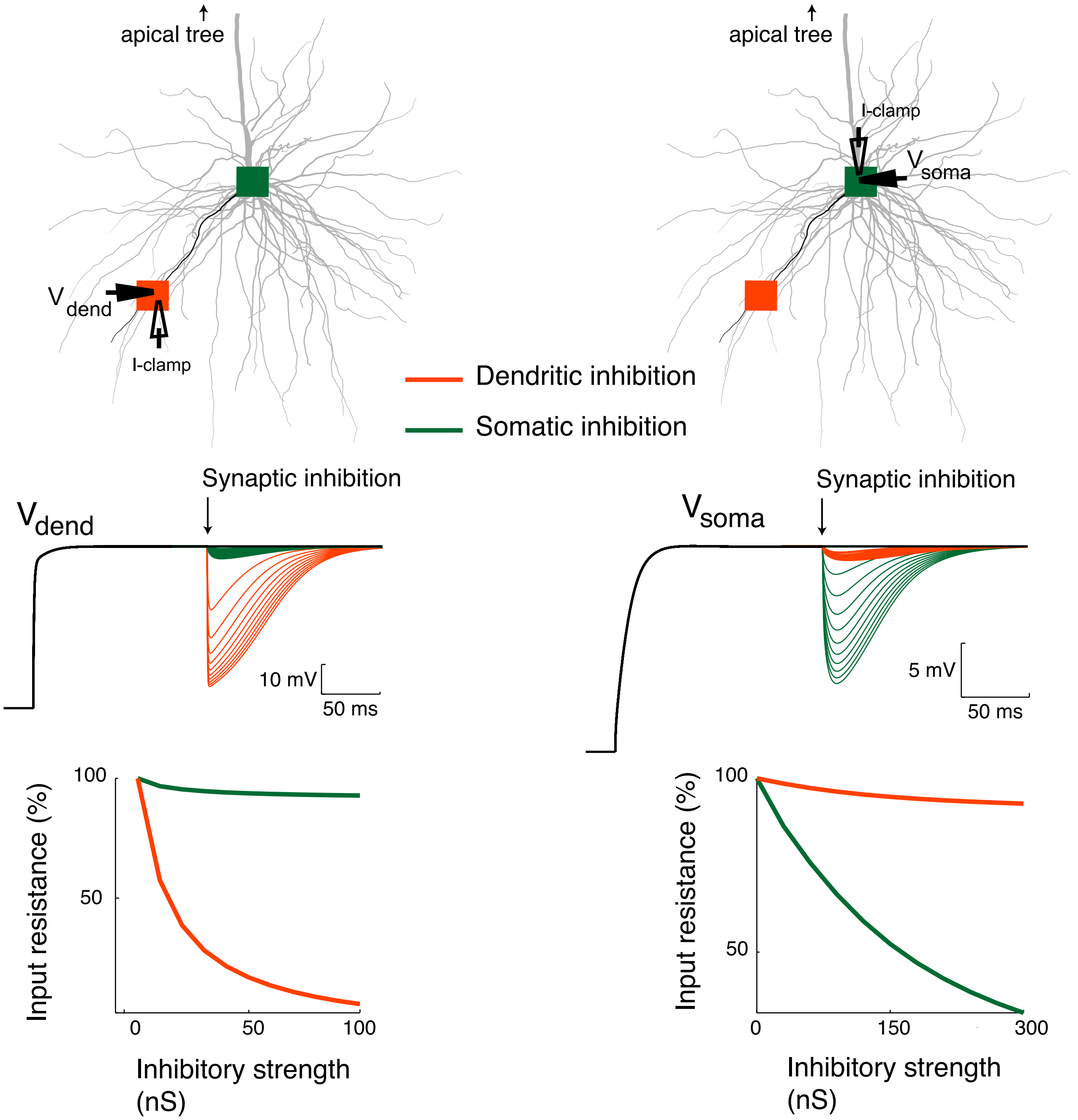

Figure S2 Experiments in the detailed compartmental model to measure input resistance changes at the somatic and dendritic location of inhibition. Inhibitory conductances of increasing strength were activated under current clamp at the soma and the dendritic location. The peak input resistance was measure as the ratio of membrane potential trough and the clamp current. Note that both the X and Y axes for the input resistance graphs on the bottom are dissimilar.
