# Supplementary material for: Location-Dependent Effects of Inhibition on Local Spiking in Pyramidal Neuron Dendrites
Source: PLoS Comput Biol. 2012 Jun 14;8(6):e1002550. doi: 10.1371/journal.pcbi.1002550 (PMC3375251; doi:10.1371/journal.pcbi.1002550)
Supplement: Figure S3 — Model of NMDA channel and definitions of NMDA spike threshold and height. (A) A single compartment model of neural membrane with an NMDA and leak conductance. (B) Example time courses of peak NMDA conductance for different values of Nsyn. Asterisk indicates time at which p(t) = 1. (C) I–V curves for leak (IL) and steady-state (i.e. p(t) = 1) NMDA conductance (INMDA) in the single compartment. Mathematical formulation of NMDA conductance shows a dependence on both membrane voltage and time-dynamics [54]. Also shown are the equations for steady-state value of Vm. The I–V plots show the resulting Vm (colored dots) for the different values of Nsyn shown in B: it is the voltage for which net inward current (INMDA) is balanced by the outward current (IL). These I–V plots can also give us an idea of how the membrane voltage will change as p(t) changes in time, as shown in B. They can be thought of as instantaneous I–V curves giving an estimate for Vm (as described above) for different values of Nsyn×p(t). The “Time” arrow follows the I–V curves for INMDA as Nsyn×p(t) changes in time. The intersection of each of these time-changing I–V curves with the leak I–V curve (IL, reflected in green) gives an estimate for Vm as a function of time. Notice that as Nsyn×p(t) changes follow the yellow curve in B, the intersection of resulting I–V curves with the leak I–V curve is a linear progression similar to the linear rise and fall of Vm in D (yellow curve). When Nsyn×p(t) changes follow the red curve in B, the intersection of resulting I–V curves with the leak I–V curve involves a non-linear jump (yellow dot and red dot) leading to the non-linear rise and fall of Vm in D (red curve). Any value of Nsyn larger than this will ensure a non-linear jump in the I–V domain (all curves below red) and consequently the time domain (D, all curves above red). Thus, spikes in the time domain correspond to all the NMDA I–V curves in C whose negative slope region lies completely below the -IL (gree [file pcbi.1002550.s003.pdf]

Figure S3, related to figures 3,4

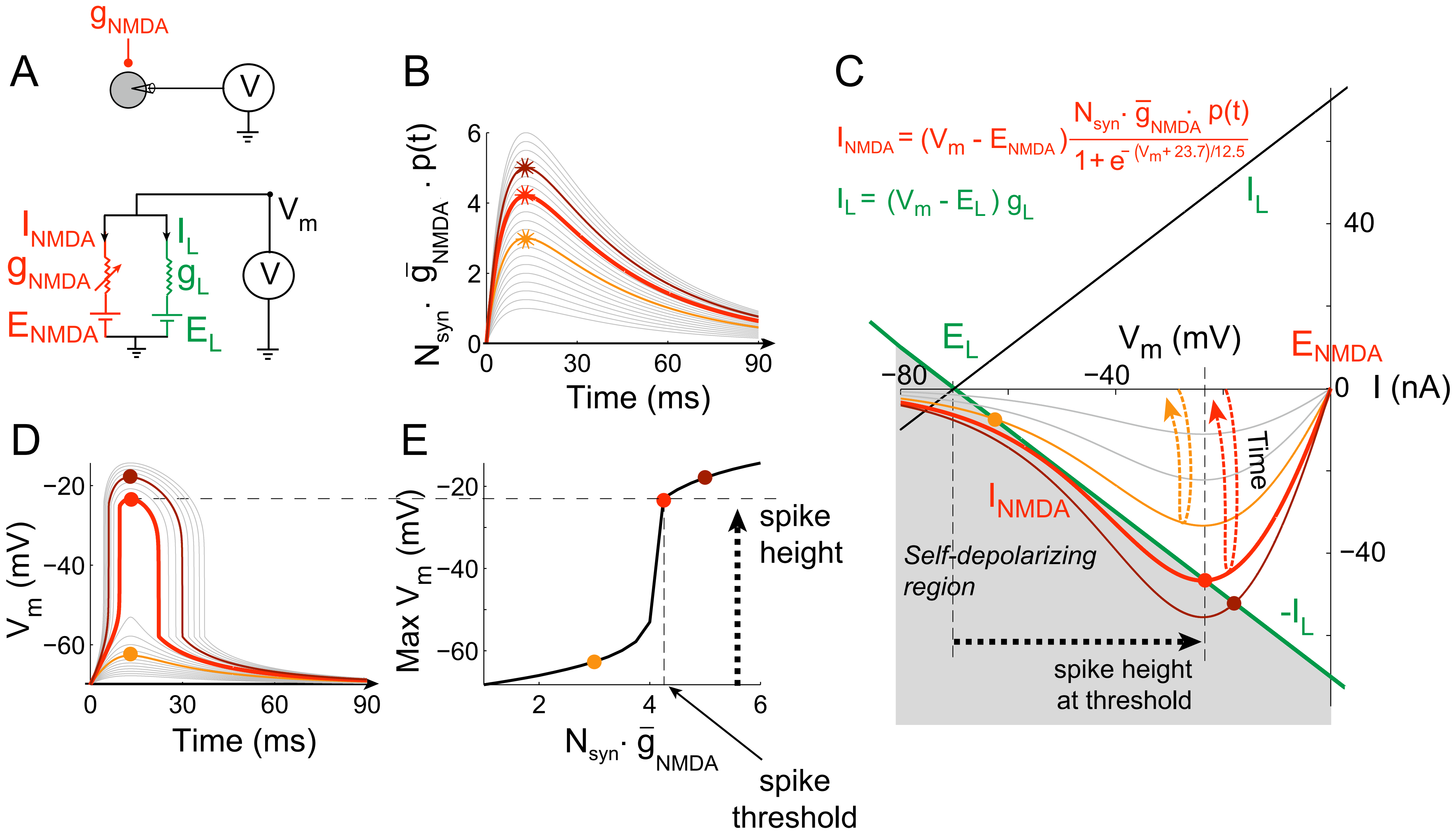

**Figure S3** Model of NMDA channel and definitions of NMDA spike threshold and height. (A) A single compartment model of neural membrane with an NMDA and leak conductance. (B) Example time courses of peak NMDA conductance for different values of $N_{syn}$. Asterisk indicates time at which p(t) = 1. (C) I-V curves for leak ($I_L$) and steady-state (i.e. p(t) = 1) NMDA conductance ($I_{NMDA}$) in the single compartment. Mathematical formulation of NMDA conductance shows a dependence on both membrane voltage and time-dynamics [54]. Also shown are the equations for steady-state value of $V_m$. The I-V plots show the resulting $V_m$ (colored dots) for the different values of $N_{syn}$ shown in B: it is the voltage for which net inward current ($I_{NMDA}$) is balanced by the outward current ($I_L$). These I-V plots can also give us an idea of how the membrane voltage will change as p(t) changes in time, as shown in B. They can be thought of as instantaneous I-V curves giving an estimate for $V_m$ (as described above) for different values of $N_{syn} \times p(t)$. The "Time" arrow follows the I-V curves for $I_{NMDA}$ as $N_{syn} \times p(t)$ changes in time. The intersection of each of these time-changing I-V curves with the leak I-V curve ($I_L$, reflected in green) gives an estimate for $V_m$ as a function of time. Notice that as $N_{syn} \times p(t)$ changes follow the yellow curve in B, the intersection of resulting I-V curves with the leak I-V curve is a linear progression similar to the linear rise and fall of $V_m$ in D (yellow curve). When $N_{syn} \times p(t)$ changes follow the red curve in B, the sintersection of resulting I-V curves with the leak I-V curve involves a non-linear jump (yellow dot and red dot) leading to the non-linear rise and fall of $V_m$ in D (red curve). Any value of $N_{syn}$ larger than this will ensure a non-linear jump in the I-V domain (all curves below red) and consequently the time domain (D, all curves above red). Thus, spikes in the time domain correspond to all the NMDA I-V curves in C whose negative slope region lies completely below the $-I_L$ (green) I-V curve, as shown. Specifically, the smallest $N_{syn}$ (red curve) for which this is the case is a representative of the minimum conductance required, given the membrane leak, to generate a spike in time-domain. The exact $N_{syn}$ would depend on the exact time-course of the conductance and the membrane capacitance.

(D) $V_m$ at the single compartment in the presence of time-varying NMDA conductance, as described in C. Sufficient value of $N_{syn}$ (red) leads to a non-linear jump in peak $V_m$ - the NMDA spike. (E). I/O curve for peak values of voltage traces shown in D. Notice the NMDA-based super-linear jump in the function.
