# Supplementary material for: Location-Dependent Effects of Inhibition on Local Spiking in Pyramidal Neuron Dendrites
Source: PLoS Comput Biol. 2012 Jun 14;8(6):e1002550. doi: 10.1371/journal.pcbi.1002550 (PMC3375251; doi:10.1371/journal.pcbi.1002550)
Supplement: Figure S4 — I–V curves at the soma in a 2-compartment model. Line colors are the same as in Figure 5B. (A) Somatic I–V curves in case of dendritic inhibition. Analysis of spike threshold and height is similar to that of Figures 5 and S3, except voltage attenuation from dendritic compartment where NMDA spike is generated, to somatic compartment where voltages are measured, leads to same horizontal compression of both NMDA (red) I–V curves. In particular, steep negative slope sections of NMDA I–V curves, and corresponding spike thresholds (red dots) are pushed proportionally closer to rest, reflecting the attenuated view of the dendritic NMDA spike at the soma (same attenuation with and without inhibition). (B) Somatic I–V curves in case of somatic inhibition. Consistent scaling of NMDA and leak I–V curves, as occurs in the dendritic compartment (Figure 4A), is disrupted from the perspective of the somatic compartment: total leak conductances scales with increasing somatic inhibition (green line), but NMDA I–V curve does not because voltage attenuation (and corresponding horizontal compression of the NMDA I–V curve) is greater with than without somatic inhibition. (PDF) [file pcbi.1002550.s004.pdf]

Figure S4, related to figure 4

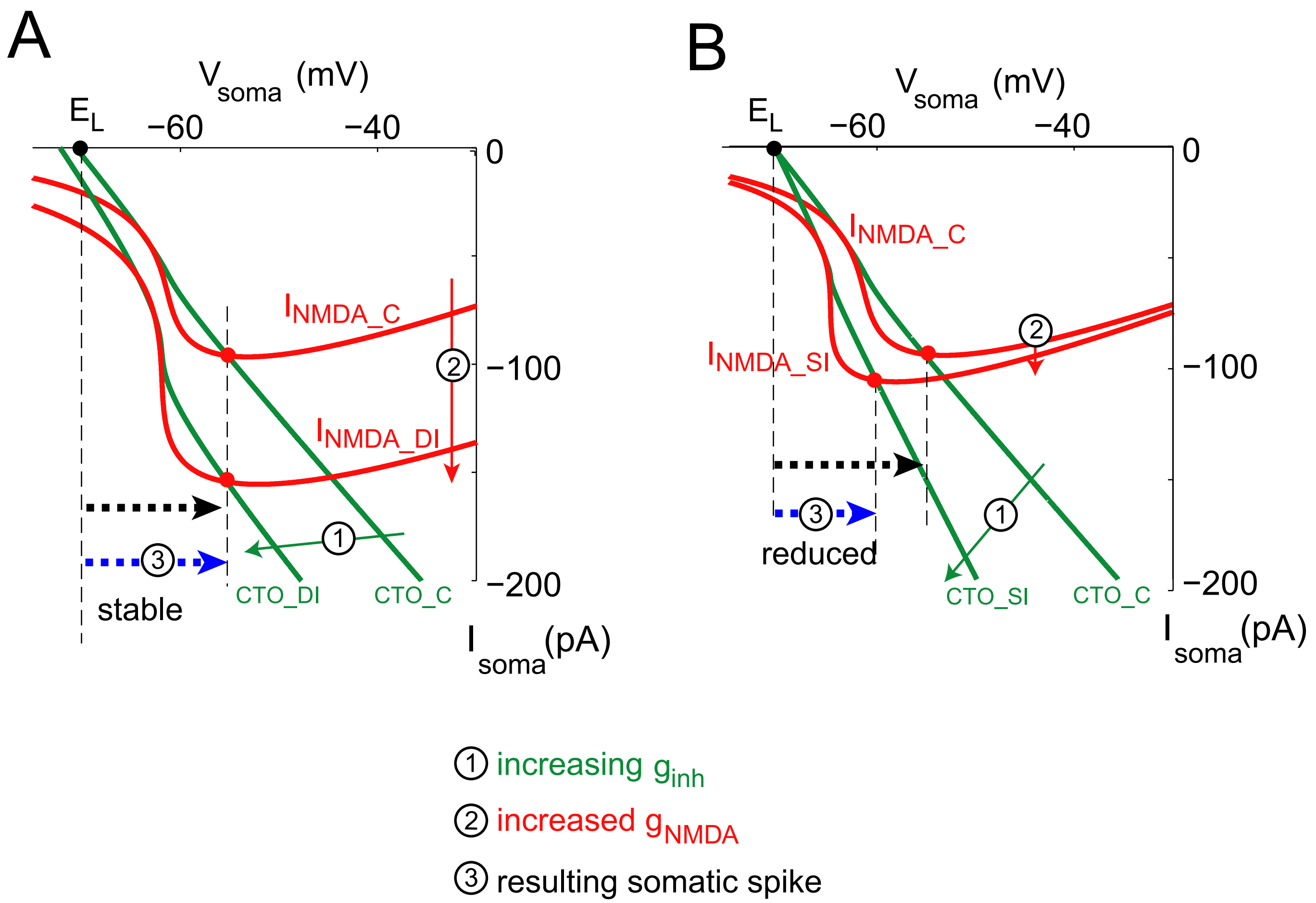

Figure S4 I-V curves at the soma in a 2-compartment model. Line colors are the same as in Figure 5B. (A) Somatic I-V curves in case of dendritic inhibition. Analysis of spike threshold and height is similar to that of Figures 5 and S3, except voltage attenuation from dendritic compartment where NMDA spike is generated, to somatic compartment where voltages are measured, leads to same horizontal compression of both NMDA (red) I-V curves. In particular, steep negative slope sections of NMDA I-V curves, and corresponding spike thresholds (red dots) are pushed proportionally closer to rest, reflecting the attenuated view of the dendritic NMDA spike at the soma (same attenuation with and without inhibition). (B) Somatic I-V curves in case of somatic inhibition. Consistent scaling of NMDA and leak I-V curves, as occurs in the dendritic compartment (Figure 4A), is disrupted from the perspective of the somatic compartment: total leak conductances scales with increasing somatic inhibition (green line), but NMDA I-V curve does not because voltage attenuation (and corresponding horizontal compression of the NMDA I-V curve) is greater with than without somatic inhibition.
