# Supplementary material for: Location-Dependent Effects of Inhibition on Local Spiking in Pyramidal Neuron Dendrites
Source: PLoS Comput Biol. 2012 Jun 14;8(6):e1002550. doi: 10.1371/journal.pcbi.1002550 (PMC3375251; doi:10.1371/journal.pcbi.1002550)
Supplement: Figure S5 — Effect of reversal potential of the inhibitory conductance on the location effect. I/O curves for somatic inhibition (A,B) and dendritic inhibition (C,D) for 3 levels of inhibitory reversal potential: −60 mV (dashed), −70 mV(solid), −80 mV (dotted). The resting membrane potential was −70 mV in both the 2-compartment (A,C) and the detailed compartmental model (B,D). (PDF) [file pcbi.1002550.s005.pdf]

Figure S5, related to figure 3

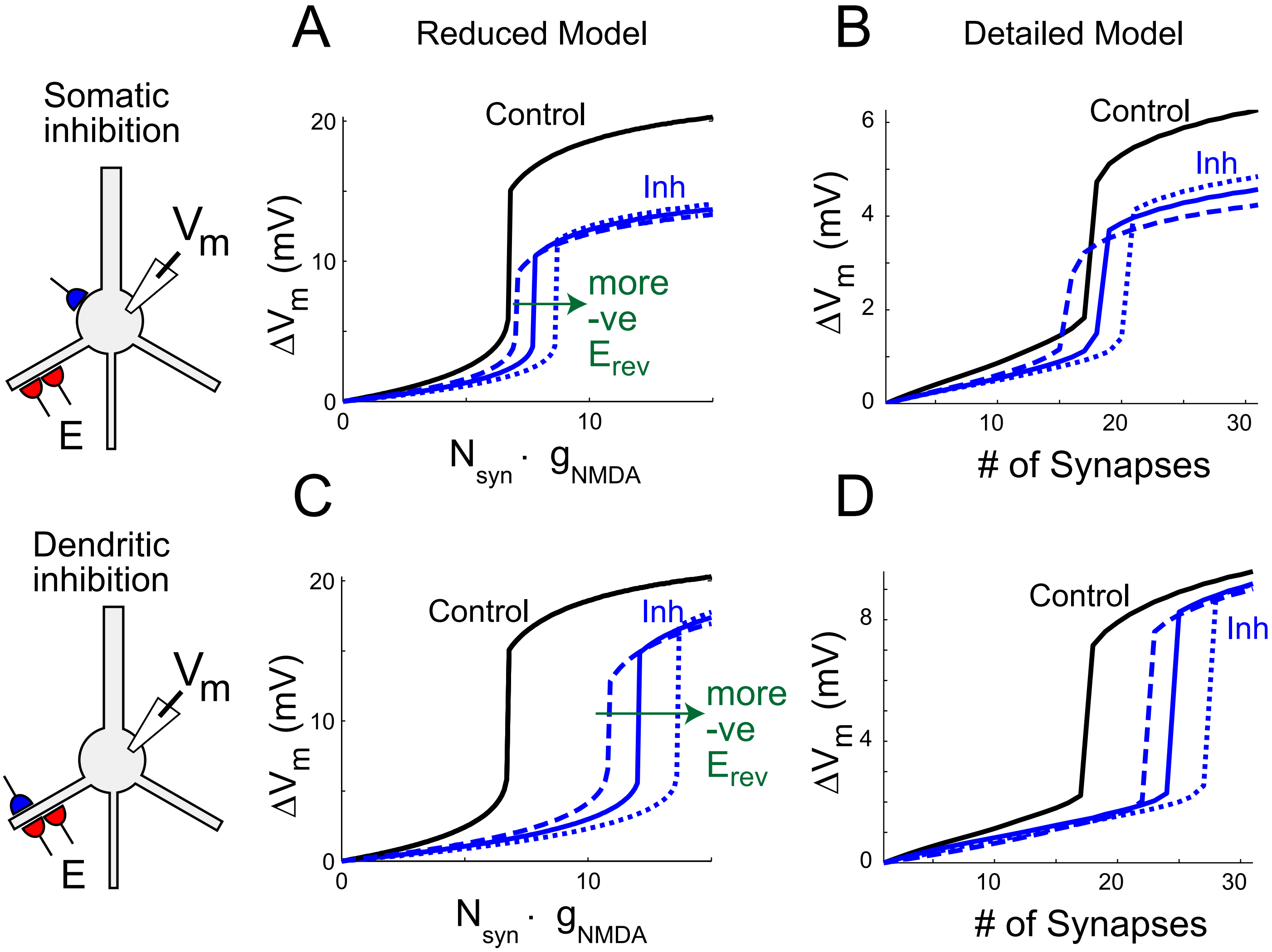

Figure S5 Effect of reversal potential of the inhibitory conductance on the location effect. I/O curves for somatic inhibition (A,B) and dendritic inhibition (C,D) for 3 levels of inhibitory reversal potential: -60 mV (dashed), -70 mV(solid), -80 mV (dotted). The resting membrane potential was -70 mV in both the 2-compartment (A,C) and the detailed com
