# Supplementary material for: Location-Dependent Effects of Inhibition on Local Spiking in Pyramidal Neuron Dendrites
Source: PLoS Comput Biol. 2012 Jun 14;8(6):e1002550. doi: 10.1371/journal.pcbi.1002550 (PMC3375251; doi:10.1371/journal.pcbi.1002550)
Supplement: Figure S6 — A conceptual model for location effect of inhibition in the presence of dendritic NMDA spikes. Assuming firing rates are determined as an average of synaptic activity over a few hundred milliseconds, this can be modeled as an averaging/pooling operation (B) over the single pulse subthreshold response (A). Coupled with a threshold-linear function for the somatic spiking mechanism, we get the predictions for suprathreshold i/o curves for both somatic and dendritic location of inhibition (C). (D) shows firing rates as a function of excitatory synapses based on simulations in a detailed compartmental model. The inhibitory synapses were either co-localized with the dendritic excitation or placed at the soma. (PDF) [file pcbi.1002550.s006.pdf]

Figure S6, related to figure 3

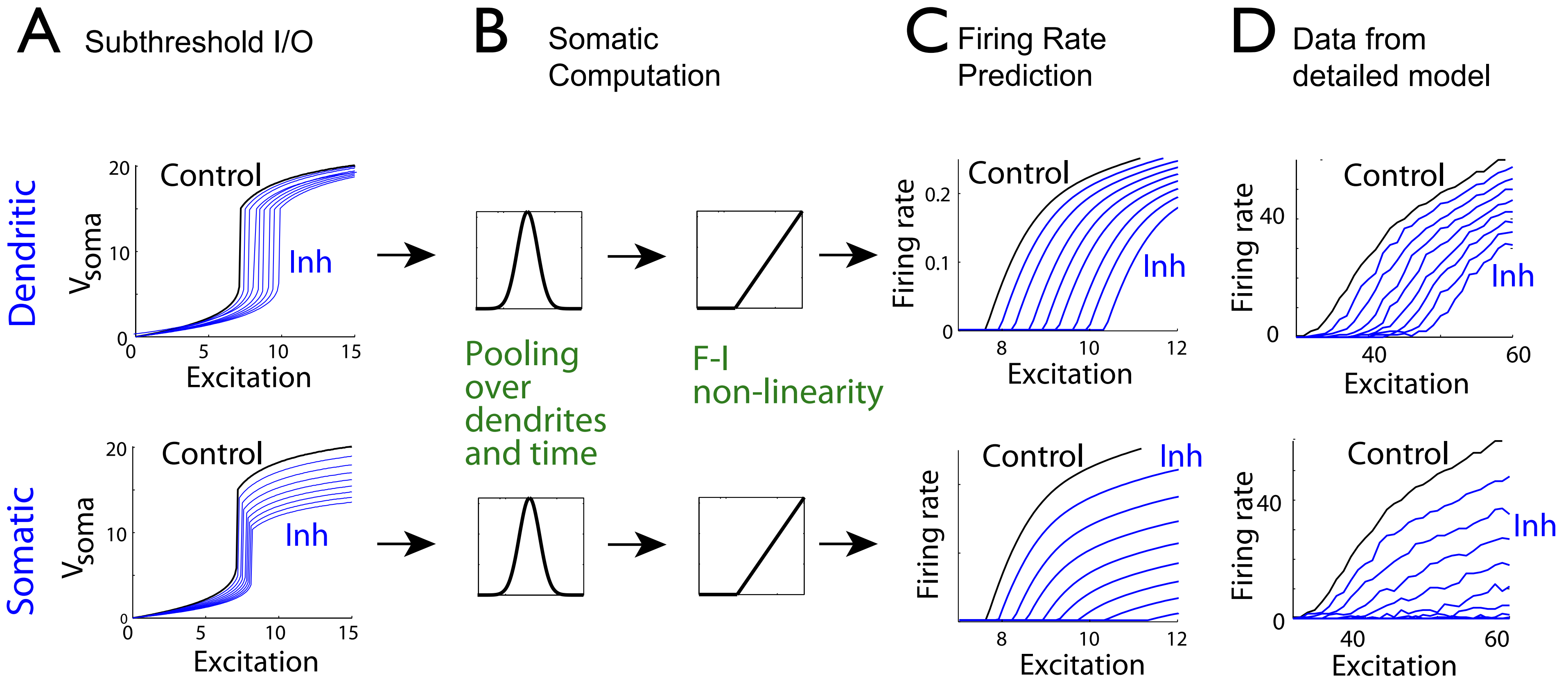

Figure S6 A conceptual model for location effect of inhibition in the presence of dendritic NMDA spikes. Assuming firing rates are determined as an average of synaptic activity over a few hundred milliseconds, this can be modeled as an averaging/pooling operation (B) over the single pulse subthreshold response (A). Coupled with a threshold-linear function for the somatic spiking mechanism, we get the predictions for suprathreshold i/o curves for both somatic and dendritic location of inhibition (C). (D) shows firing rates as a function of excitatory synapses based on simulations in a detailed compartmental model. The inhibitory synapses were either co-localized with the dendritic excitation or placed at the soma.
