# Supplementary material for: Location-Dependent Effects of Inhibition on Local Spiking in Pyramidal Neuron Dendrites
Source: PLoS Comput Biol. 2012 Jun 14;8(6):e1002550. doi: 10.1371/journal.pcbi.1002550 (PMC3375251; doi:10.1371/journal.pcbi.1002550)
Supplement: Figure S7 — Sensitivity of the location effect to the onset and duration of inhibition. (A) Representative location of excitatory (red square) and inhibitory (blue/dark green/light green circle) synapses in the morphologically detailed model. (B) I/O function between peak membrane potential and strength of excitation for the case of no inhibition (black), slow (blue), fast (dark green), and delayed fast (light green) inhibition in a detailed compartmental model. For the cases shown here, the peak inhibitory conductance was 4 nS for dendritic inhibition and 24 nS for somatic inhibition. (C) Summary of the effect of inhibition offset and duration at the somatic (triangles) and dendritic location (circles) in the detailed model, expressed as joint % change in spike height and threshold relative to no-inhibition control (black square at the origin). Peak conductance for dendritic inhibition cases shown here was 4, 8, 12, 16 and 20 nS, while that for somatic inhibition was 24, 48, 72, 96 and 120 nS. Each excitatory synapse in this experiment had 6 nS peak AMPA conductance. Excitatory synapses with 1.5 nS peak AMPA conductance with similar distribution of density along the dendrite gave similar results. Red and green shaded areas were carried over from Figure 2 to indicate general trends for dendritic vs. somatic inhibition in the in vitro data. (PDF) [file pcbi.1002550.s007.pdf]

Figure S7 Jadi

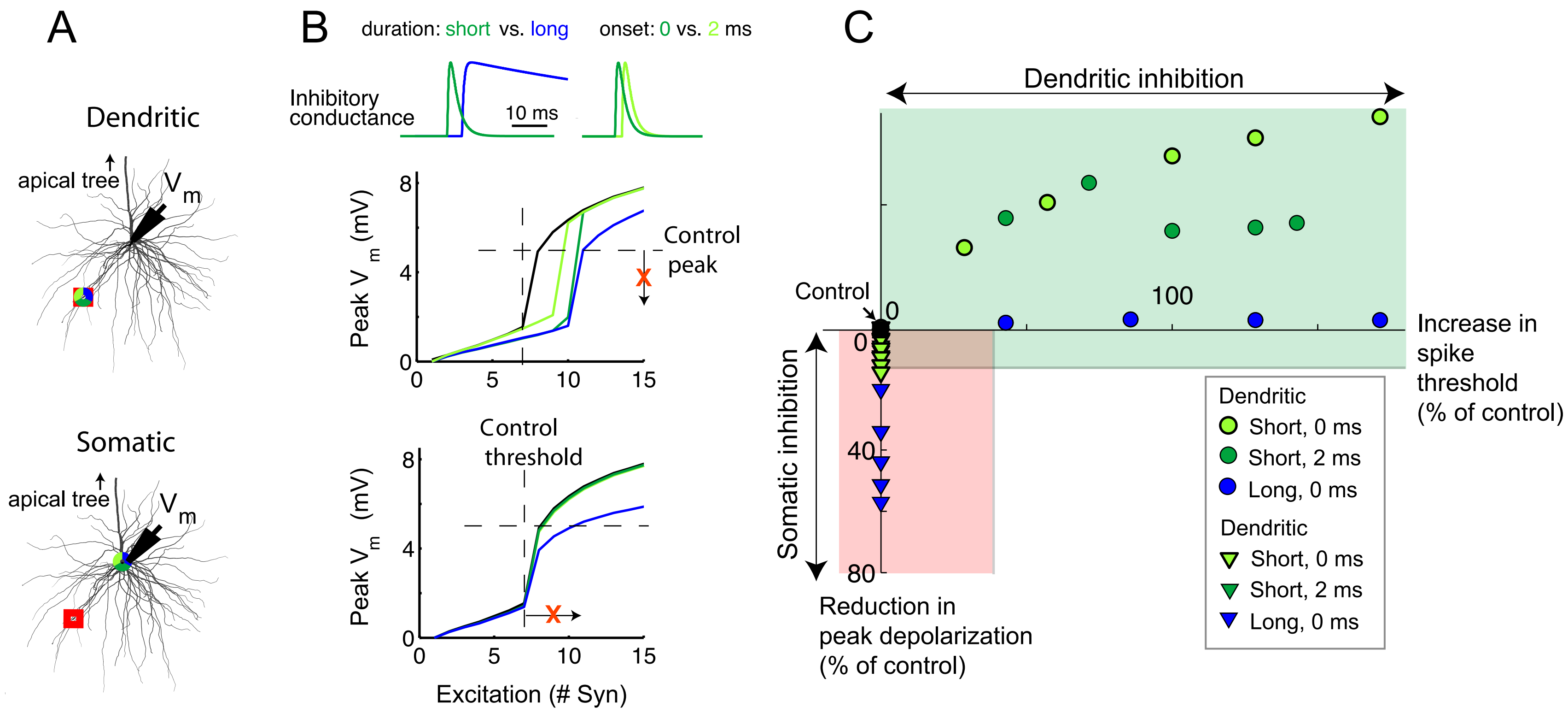

Figure S7 Sensitivity of the location effect to the onset and duration of inhibition. (A) Representative location of excitatory (red square) and inhibitory (blue/dark green/ light green circle) synapses in the morphologically detailed model. (B) I/O function between peak membrane potential and strength of excitation for the case of no inhibition (black), slow (blue), fast (dark green), and delayed fast (light green) inhibition in a detailed compartmental model. For the cases shown here, the peak inhibitory conductance was 4 nS for dendritic inhibition and 24 nS for somatic inhibition. (C) Summary of the effect of inhibition offset and duration at the somatic (triangles) and dendritic location (circles) in the detailed model, expressed as joint % change in spike height and threshold relative to no-inhibition control (black square at the origin). Peak conductance for dendritic inhibition cases shown here was 4, 8, 12, 16 and 20 nS, while that for somatic inhibition was 24, 48, 72, 96 and 120 nS. Each excitatory synapse in this experiment had 6 nS peak AMPA conductance. Excitatory synapses with 1.5 nS peak AMPA conductance with similar distribution of density along the dendrite gave similar results. Red and green shaded areas were carried over from Fig. 2 to indicate general trends for dendritic vs. somatic inhibition in the in vitro data.
