# Supplementary material for: Location-Dependent Effects of Inhibition on Local Spiking in Pyramidal Neuron Dendrites
Source: PLoS Comput Biol. 2012 Jun 14;8(6):e1002550. doi: 10.1371/journal.pcbi.1002550 (PMC3375251; doi:10.1371/journal.pcbi.1002550)
Supplement: Figure S8 — Comparison of divisive (green) and subtractive (blue) prediction for the computational effect of somatic inhibition on somatic membrane potential in the detailed model. Red squares are data points from simulation of the detailed compartmental model (also shown in Figure 2). The asterisk indicates the data point used for generating the prediction. (PDF) [file pcbi.1002550.s008.pdf]

Figure S8 Jadi

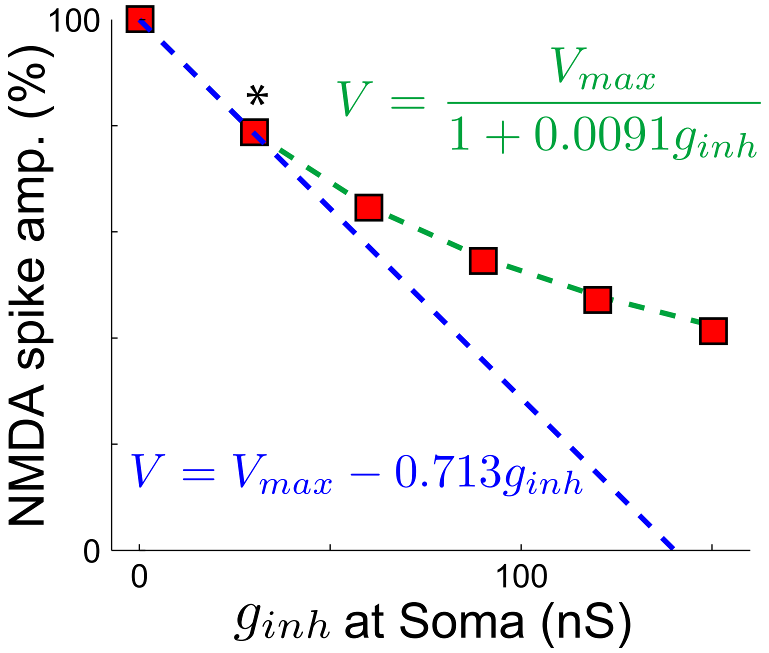

Figure S8 Comparison of divisive (green) and subtractive (blue) prediction for the computational effect of somatic inhibition on somatic membrane potential in the detailed model. Red squares are data points from simulation of the detailed compartmental model (also shown in Figure 2). The asterisk indicates the data point used for generating the prediction.
