# Supplementary material for: Location-Dependent Effects of Inhibition on Local Spiking in Pyramidal Neuron Dendrites
Source: PLoS Comput Biol. 2012 Jun 14;8(6):e1002550. doi: 10.1371/journal.pcbi.1002550 (PMC3375251; doi:10.1371/journal.pcbi.1002550)
Supplement: Text S1 — Explanation for constancy of NMDA spike height with I–V plots. (PDF) [file pcbi.1002550.s009.pdf]

S1. **Constancy of NMDA spike height explained by I-V plots**

As described in Methods and illustrated in the I-V plots in Figure 5B, for a given net leak conductance g_L in a compartment, the I-V curve for the net leak conductance (green) and the NMDA conductance (red) meet at exactly two points for threshold condition; they intersect at one point (red dot), while they are tangential at the other (In contrast, I-V curves for g_L and subthreshold NMDA conductances meet at one or three points, while those for suprathreshold conductances meet at only one point. See Figure S3C). The tangency is the geometric criterion for NMDA conductance to be considered at spike threshold, while the intersection point (red dot) is the value of steady state membrane potential (NMDA spike height) at the threshold. Both points on the I-V plots are the solutions to equation (2) (restated here) for threshold value of $N_{syn} = N_{syn\theta}$:

$$V_m = \frac{E_L}{\left(\frac{N_{syn\theta}}{g_L} \times \frac{\bar{g}_{NMDA}}{1 + e^{-(V_m + 7)/12.5}} + 1 \right)}$$

If the net leak conductance g_L in the compartment now increases by a factor s, then as long as $N_{syn\theta}$ also scales by s, the nature as well as the values of solution to the above equation will remain essentially the same. Specifically, it will consist of exactly two meeting points for the I-V plots, which means the scaled NMDA channels ($s \times N_{syn\theta}$) will also be the threshold value for generating NMDA spike in the presence of new leak $s \times g_L$. Also, the value of V_m at the intersection

point (red dots in Fig 4B), which corresponds to the spike height at threshold, will remain constant for equal scaling of g_L and $N_{syn}\theta$. This is the basis of a constant NMDA spike height in a single compartment for any level of net leak conductance.
